# Dual-action nanotheranostics targeting cell-free DNA rescues aberrant alternative polyadenylation to ameliorate periodontitis

**DOI:** 10.1016/j.mtbio.2026.103776

**Published:** 2026-10-31

**Authors:** Bin Gu, Shuyuan Qu, Yutong Wei, Yilong Zhao, Shuchang Hu, Xiaohan Zeng, Yaoting Ji, Qin Zhao

**Affiliations:** State Key Laboratory of Oral & Maxillofacial Reconstruction and Regeneration, Key Laboratory of Oral Biomedicine Ministry of Education, Hubei Key Laboratory of Stomatology, School & Hospital of Stomatology, Wuhan University, Wuhan, 430079, China

**Keywords:** Periodontitis, Alternative polyadenylation, 3′UTR, Cell-free DNA, Nano materials

## Abstract

While periodontal pathogens (*Porphyromonas gingivalis*) are known to severely disrupt macrophage post-transcriptional homeostasis, the precise extracellular instigator remains elusive. Here, through comparative transcriptomic profiling, we identify cell-free DNA (cfDNA) as this fundamental pathogenic trigger. Comprehensive bioinformatic analysis uncovers that cfDNA governs macrophage fate by fundamentally rewiring the global alternative polyadenylation (APA) equilibrium. Specifically, cfDNA forces pro-inflammatory (M1) transcripts into translationally hyperactive short-3′-untranslated region (3′UTR) isoforms, while simultaneously suppressing anti-inflammatory (M2) programs via 3′UTR lengthening. To therapeutically intercept this cfDNA-APA pathogenic axis, we engineered a homologous nanotheranostic platform (DA-APAT). Addressing the unique clinical demands for early periodontitis screening, its polycationic PEI shell enables highly sensitive salivary cfDNA theranostic monitoring. Crucially, this monitoring readout directly calibrates the in situ enzymatic clearance load executed by the internally encapsulated DNase I. Synergized by the intrinsic reactive oxygen species (ROS) scavenging capability of the hollow mesoporous cerium oxide core, this monitoring nanoplatform decisively silences intracellular DNA-sensing pathways (cGAS-STING/TLR9), rectifies the disrupted APA, and contributes robust M2 macrophage repolarization. In an orthotopic murine periodontitis model, this nanotherapy effectively halts alveolar bone loss and facilitates profound periodontal regeneration with excellent biocompatibility. This work unveils a cfDNA-driven RNA regulatory paradigm in immune dysregulation and provides a monitoring precision nanotheranostic modality for inflammatory therapy.

## Graphical abstract

**Figure:**
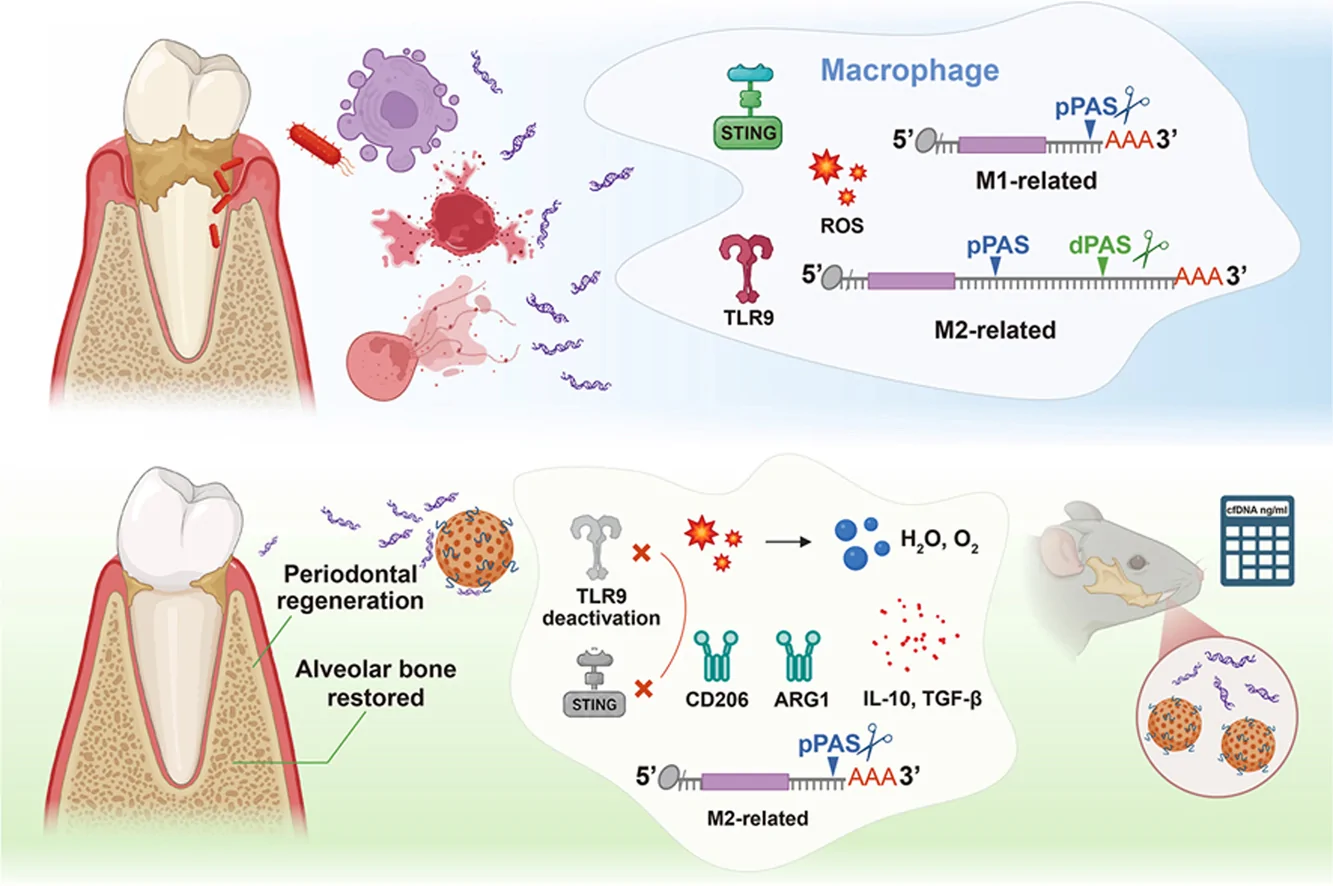

## Teaser

**Synopsis:** Extracellular cell-free DNA (cfDNA) drives periodontitis by pathogenically rewiring the macrophage alternative polyadenylation (APA) landscape. To intercept this, we engineered a homologous nanotheranostic platform (DA-APAT). By enabling highly sensitive salivary cfDNA diagnostics to guide precise in situ enzymatic clearance, DA-APAT thoroughly rectifies the APA equilibrium and orchestrates robust periodontal regeneration.

## 1 Introduction

Periodontitis is a chronic, tissue-destructive inflammatory disease driven by the dysbiosis of dental plaque biofilms—with *Porphyromonas gingivalis* (*P. gingivalis*) acting as a keystone pathogen—and the subsequent aberrant host immune responses [1], [2], [3]. Under continuous pathogen invasion, stromal and resident immune cells release a plethora of pro-inflammatory factors and chemokines, triggering a massive influx of neutrophils and monocytes/macrophages. The activated immune cells subsequently release neutrophil extracellular traps (NETs), pro-inflammatory cytokines, and tissue-destructive enzymes (e.g., nitric oxide synthase), thereby exacerbating tissue damage [4], [5], [6], [7]. This vicious cascade systematically amplifies inflammatory signaling and primes adaptive immunity, culminating in persistent immune dysregulation [8], [9], [10], [11], [12]. Consequently, traditional clinical interventions, such as pure antibacterial treatments and supragingival scaling, are largely inadequate. More importantly, there is a critical lack of early diagnostic modalities and targeted therapies against initial pathogenic invasion. Thus, developing an effective theranostic strategy is of profound clinical significance.

Within the intense host-pathogen battleground of periodontal pockets, massive cell death (such as NETosis in neutrophils and pyroptosis in macrophages) leads to the release and extracellular accumulation of cell-free DNA (cfDNA) [13], [14]. Increasing evidence indicates that NET-associated DNA represents an important source of circulating extracellular DNA, highlighting the dynamic relationship between neutrophil activation, extracellular DNA release, and cfDNA homeostasis [15]. Rather than being merely a by-product of cellular injury, cfDNA can exert potent immunomodulatory effects in inflammatory microenvironments [15], [16], [17], [18]. Extracellular DNA may function as damage-associated molecular patterns (DAMP) and activate DNA-sensing pathways, including TLR9 and cGAS-STING, thereby promoting inflammatory cytokine production and sustaining tissue injury [19]. Consistent with this concept, both local and circulating cfDNA levels have been reported to increase in periodontitis and to associate with disease severity [20], [21], [22]. Importantly, therapeutic depletion of extracellular DNA has also shown potential for attenuating periodontal inflammation, suggesting that cfDNA is not merely a disease-associated biomarker but may actively participate in inflammatory pathogenesis [21].

Beyond its biological activity, cfDNA has emerged as a powerful source of molecular information for non-invasive disease assessment. Pioneering work by Dennis Lo and colleagues established the clinical potential of circulating cell-free DNA, beginning with the discovery of fetal DNA in maternal plasma and subsequently extending to cancer-associated circulating DNA and liquid biopsy applications [23], [24]. More recent studies have demonstrated that cfDNA contains information beyond sequence alterations, with DNA methylation, nucleosome positioning, fragment-size distributions, fragmentation patterns, and fragment-end characteristics providing information about tissue of origin and disease state [25], [26]. In particular, work from Kun Sun and colleagues has established cfDNA fragmentation profiles as a rich molecular feature for inferring tissue origin and improving cancer detection, while studies from Alain Thierry and colleagues have further highlighted the contribution of NET-associated DNA to the circulating DNA pool and the biological dynamics of extracellular DNA [25], [26]. Nevertheless, despite these advances in the diagnostic and translational applications of cfDNA, its specific physiological and pathogenic functions in inflammatory immune cells remain incompletely understood.

Alternative polyadenylation (APA) has emerged as a crucial post-transcriptional regulatory mechanism across various physiological and pathological processes, especially in immune responses [27], [28], [29]. APA determines the length and regulatory elements of the mRNA 3′-untranslated region (3′UTR), thereby governing mRNA stability, subcellular localization, and translation efficiency. Notably, highly proliferative cells, such as activated T cells, tend to express transcripts with shortened 3′UTRs, whereas a progressive lengthening of 3′UTRs is observed during murine embryonic development and C2C12 myoblast differentiation [30], [31]. Indeed, in our previous work, we uncovered that *P. gingivalis* infection severely disrupts the post-transcriptional homeostasis in the periodontitis microenvironment, compelling pro-inflammatory macrophages to extensively adopt shortened 3′UTR [32]. However, the precise extracellular instigator mediating this *P. gingivalis*-induced transcriptomic rewiring remained elusive. Intriguingly, viral remnants or cfDNA released from massive cell death in the infectious battleground may hijack this 3′-end processing machinery. Building upon our prior findings, we performed deep comparative transcriptomic analysis and remarkably discovered that direct cfDNA stimulation closely mirrors the profound pathological landscape previously observed with live P. g infection. DaPars2 analysis [33] further explicitly identified accumulated cfDNA as the potent upstream post-transcriptional rewiring agent that fundamentally reprograms the global APA landscape of macrophages. Specifically, cfDNA forces pro-inflammatory (M1) transcripts into translationally hyperactive short-3′UTR isoforms, while simultaneously suppressing anti-inflammatory (M2) programs via 3′UTR lengthening. This cfDNA-driven APA reprogramming precisely delineates the molecular missing link explaining how macrophages become permanently locked into a destructive phenotype during periodontitis.

To therapeutically intercept this cfDNA-APA pathogenic axis, the targeted depletion of cfDNA is paramount. Herein, we engineered a homologous "diagnostic-guided therapeutic" nanoplatform, termed Dual-Action APA Theranostics (DA-APAT), based on a hollow mesoporous cerium oxide (HMCeO_2_) core loaded with DNase I and coated with a polyethylenimine (PEI) shell. Unlike conventional theranostics that passively combine diagnostic imaging agents and therapeutic drugs as separate, fragmented entities, DA-APAT operates on an integrated, structure-driven feedback loop (Scheme 1). The polycationic PEI shell acts as an intrinsic cfDNA-capturing domain, endowing the system with a natural potential for non-invasive salivary cfDNA diagnostics. Concurrently, synergizing with the intrinsic reactive oxygen species (ROS) scavenging capability of the CeO_2_ nanozyme, DA-APAT thoroughly silences intracellular DNA-sensing axes (cGAS-STING and TLR9) and restores the macrophage anti-inflammatory phenotype. In a murine periodontitis model, this dual-action platform fully unleashed its theranostic potential: it enabled precise liquid biopsy via adsorbed salivary cfDNA, while orchestrating robust M2 macrophage repolarization to completely halt alveolar bone loss and promote periodontal regeneration. This work unveils a cfDNA-driven post-transcriptional paradigm in immune dysregulation and provides a logically designed nanotheranostic modality for precision inflammatory therapy.

**Scheme 1:**
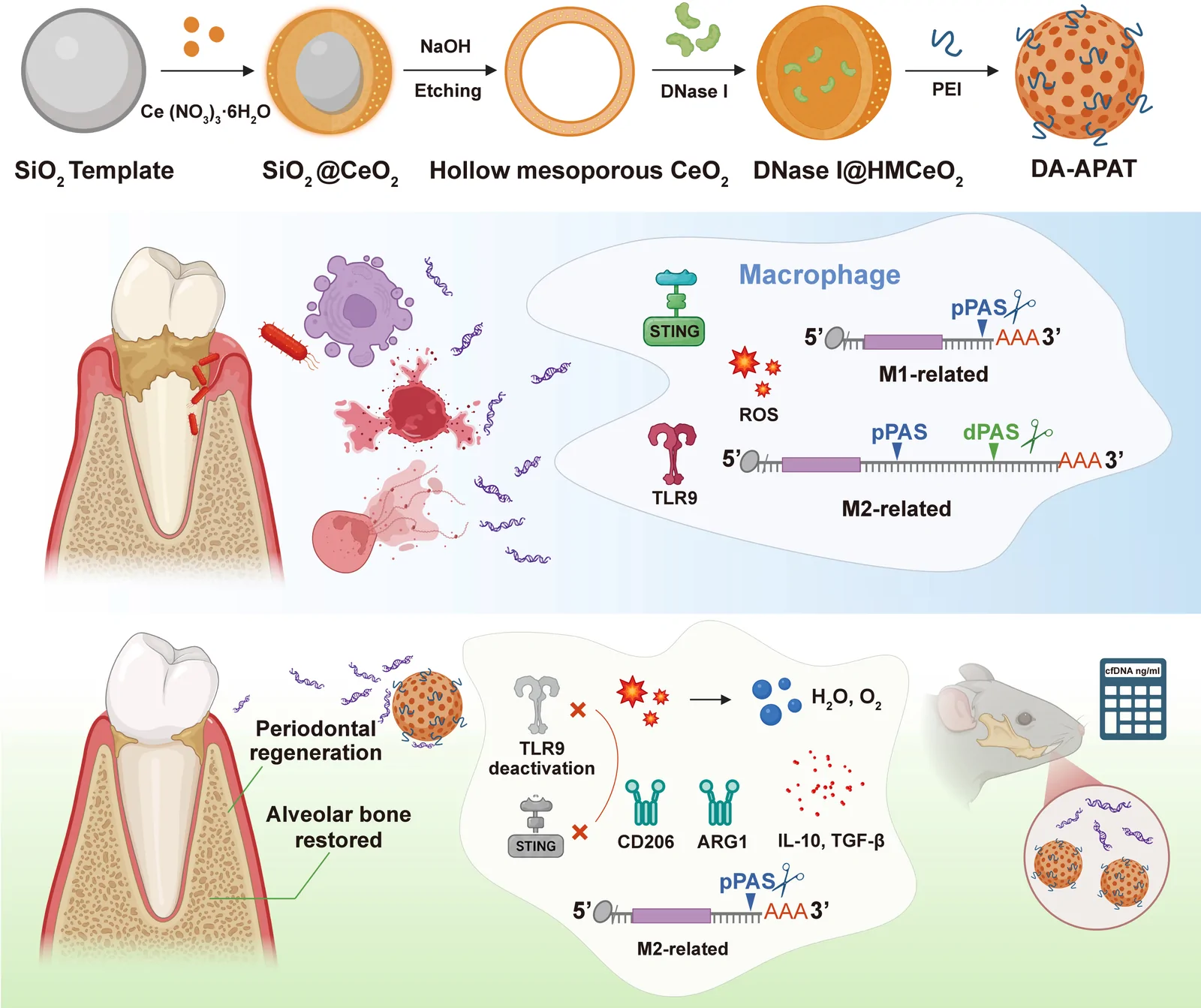
Schematic diagram of the design and function of DA-APAT.

## 2 Results

### 2.1 cfDNA acts as the key pathogenic mediator driving *P. gingivalis*-induced immune dysregulation

To elucidate the initiating molecular events underlying *P. gingivalis* (*P. g*)-induced periodontal immune dysregulation, we utilized RAW264.7 macrophages—a well-established in vitro model for studying inflammatory responses and osteoclastogenesis in periodontitis [34], [35]. Comparative transcriptomic profiling (RNA-seq) of macrophages following *P. g* stimulation revealed 2276 specifically expressed genes in the *P. g* group alongside 25,918 intersecting genes relative to the control (Fig. 1A). Quantitative analysis of the differentially expressed genes (DEGs) demonstrated a massive transcriptional shift, with 4752 genes significantly up-regulated and 4351 down-regulated (Fig. 1B), as further illustrated by the volcano plot of representative DEGs (Fig. 1C). To decode the biological implications behind this immense transcriptomic alteration, we performed Gene Ontology (GO) and Kyoto Encyclopedia of Genes and Genomes (KEGG) enrichment analyses. Strikingly, beyond classical inflammatory pathways (e.g., Immune system process, TNF, and NF-κB signaling), biological processes related to the "cellular response to DNA damage stimulus," "DNA repair," and "mRNA processing" were highly enriched (Fig. 1D and E). Crucially, Gene Set Enrichment Analysis (GSEA) revealed that the "Cytosolic DNA-sensing pathway" (Normalized Enrichment Score NES: 2.91) and the "NOD-like receptor signaling pathway" (NES: 3.20) were aberrantly activated (Fig. 1F). This unexpected finding prompted a bold hypothesis: *P. g* infection not only directly activates Toll-like receptors but also triggers massive cell death, leading to the extracellular release and accumulation of host cfDNA. Once in the microenvironment or cytosol, this cfDNA likely acts as an endogenous danger signal (DAMP) and serves as an independent, key mediator driving the pathological progression of periodontitis.

**Fig. 1:**
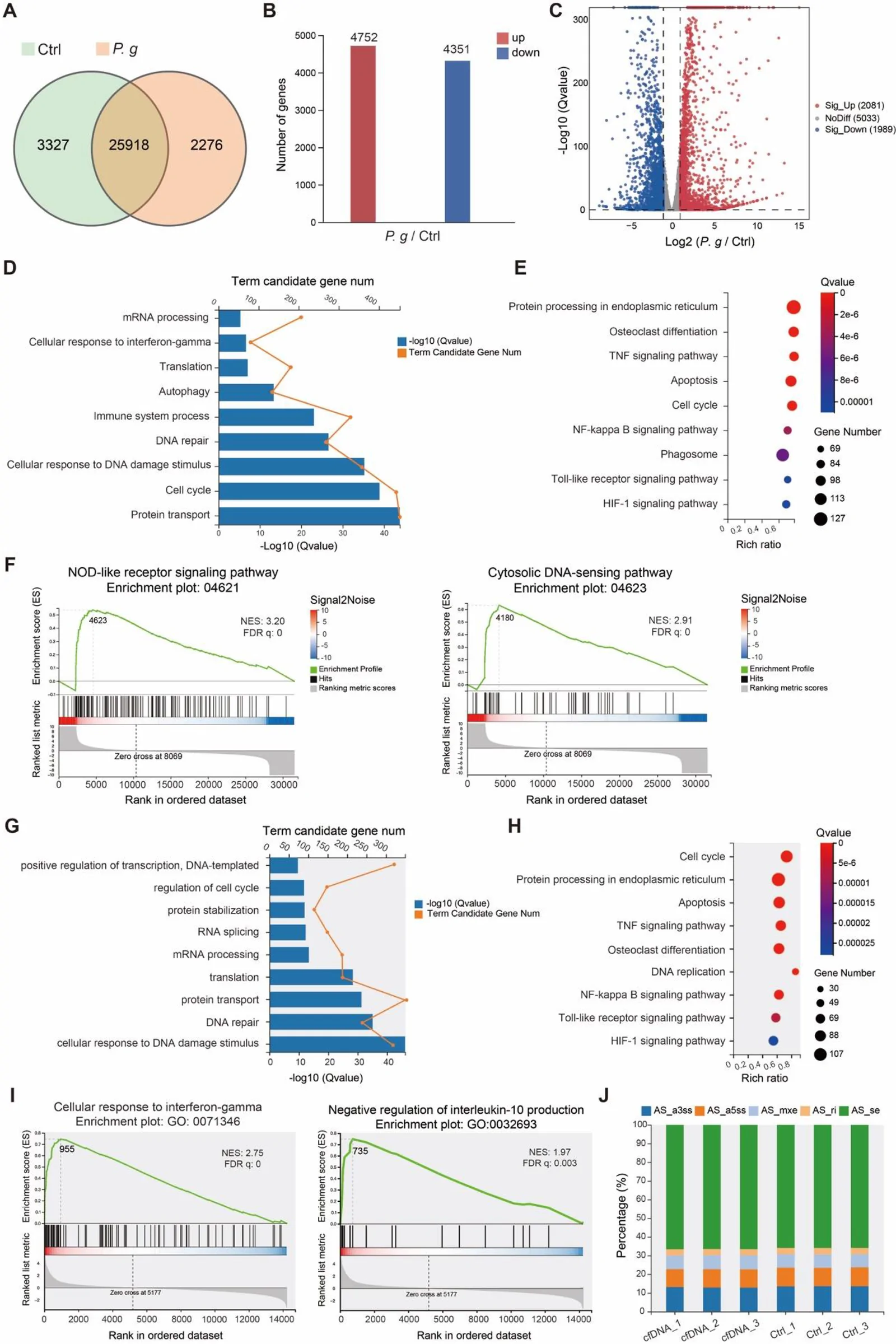
**RNA-seq analysis of RAW264.7 cells after stimulation with *P. g* and cfDNA, respectively.** (A) Venn diagram illustrating intersecting and uniquely expressed genes in RAW264.7 cells treated with *P. g* versus control. (B) Quantitative statistics of DEGs post-*P. g* infection. (C) Volcano plot of DEGs induced by *P. g* stimulation. Log2 > 1.5, q < 0.05. (D) Top enriched GO and (E) KEGG pathways based on the up-regulated genes in the *P. g* group. (F) GSEA plots revealing the hyperactivation of cytosolic DNA-sensing and NOD-like receptor signaling pathways. (G) GO and (H) KEGG pathway enrichment analyses for the cfDNA-treated group, displaying striking consistency with *P. g* infection. (I) GSEA plots demonstrating that cfDNA enhances cellular response to IFN-γ while suppressing IL-10 production. (J) Quantitative comparison of various AS events between groups.

To validate this hypothesis, we treated RAW264.7 cells with calf thymus DNA—a standard double-stranded DNA surrogate widely used to mimic cfDNA in vitro [36]—and conducted a secondary RNA-seq analysis. The volcano plot and DEG statistics revealed that cfDNA alone provoked profound transcriptional alterations, comprising 3379 up-regulated and 3110 down-regulated genes (Fig. S1). A joint analysis demonstrated that 590 genes exhibited expression patterns identical to those in the *P. g*-stimulated group, accounting for 62.6% of the total DEGs induced by cfDNA (Fig. S2). Remarkably, the transcriptomic landscape induced by cfDNA was highly congruent with that of *P. g* infection. GO and KEGG analyses confirmed that the cfDNA-treated group was analogously enriched in typical periodontitis-related pathways, including DNA damage response, DNA repair, protein processing in the endoplasmic reticulum, and TNF/NF-κB signaling (Fig. 1G and H). Phenotypically, GSEA explicitly demonstrated that cfDNA treatment not only significantly up-regulated the cellular response to interferon-gamma (IFN-γ)—a hallmark of pro-inflammatory M1 macrophages—but also negatively regulated interleukin-10 (IL-10) production, which is characteristic of anti-inflammatory M2 macrophages (Fig. 1I). These results robustly establish cfDNA as a core driving force that rewires macrophage polarization toward a destructive M1 phenotype while suppressing the M2 phenotype.

While the canonical cGAS-STING and TLR9 pathways elegantly explain the initiation of cfDNA-induced transcriptional cascades, recent cutting-edge studies indicate that innate immune sensing of nucleic acids is intricately coupled with post-transcriptional RNA processing [37], [38]. Upon massive DNA sensor activation, downstream effectors not only trigger transcription factors (e.g., NF-κB) but also induce extensive alternative splicing (AS) in this pathway. Multiple AS isoforms of cGAS, STING, and TBK1 have been found to form a positive/negative feedback loop affecting inflammatory responses [39]. Prompted by this intertwined relationship, and considering that "mRNA processing" and "RNA splicing" were recurrently enriched in our transcriptomic GO analyses (Fig. 1D and G), we hypothesized that the pathogenic role of cfDNA extends beyond mere transcriptional initiation, profoundly hijacking the post-transcriptional landscape to sustain the inflammatory state. Typically, AS is the most prevalent form of post-transcriptional regulation. However, upon rigorous quantitative profiling of various AS events (including a3ss, a5ss, mxe, ri, and se) across the groups, no significant statistical differences were observed (Fig. 1J). This paradoxical observation systematically ruled out the dominance of conventional alternative splicing, steering our focus toward other critical RNA processing mechanisms.

### 2.2 cfDNA reprograms macrophage polarization via APA

Given that conventional alternative splicing could not account for the cfDNA-induced post-transcriptional landscape, we employed the DaPars2 algorithm to deeply mine the RNA-seq data for APA events. The Percentage of Distal poly(A) site Usage Index (PDUI) was calculated to depict the global APA dynamics post-cfDNA treatment. The resulting volcano plot revealed that 510 genes underwent significant 3′UTR shortening, while 355 genes exhibited significant 3′UTR lengthening (Fig. 2A). This widespread, dynamic switching of 3′UTR unequivocally proves that cfDNA profoundly disrupts the global APA equilibrium in macrophages. Intriguingly, correlating these APA events with their biological functions unveiled a highly pathological pattern. GO enrichment analysis of the gene set featuring 3′UTR lengthening revealed a striking concentration in biological processes essential for macrophage homeostasis and anti-inflammatory functions (M2 phenotype), including "regulation of leukocyte differentiation," "regulation of DNA repair," "cellular response to macrophage colony-stimulating factor stimulus," "regulation of myeloid cell differentiation," and "antiviral innate immune response" (Fig. 2B). This foundational finding suggested that cfDNA suppresses the macrophage M2 program by forcing these anti-inflammatory transcripts into extended 3′UTR isoforms, thereby subjecting them to post-transcriptional translational repression.

**Fig. 2:**
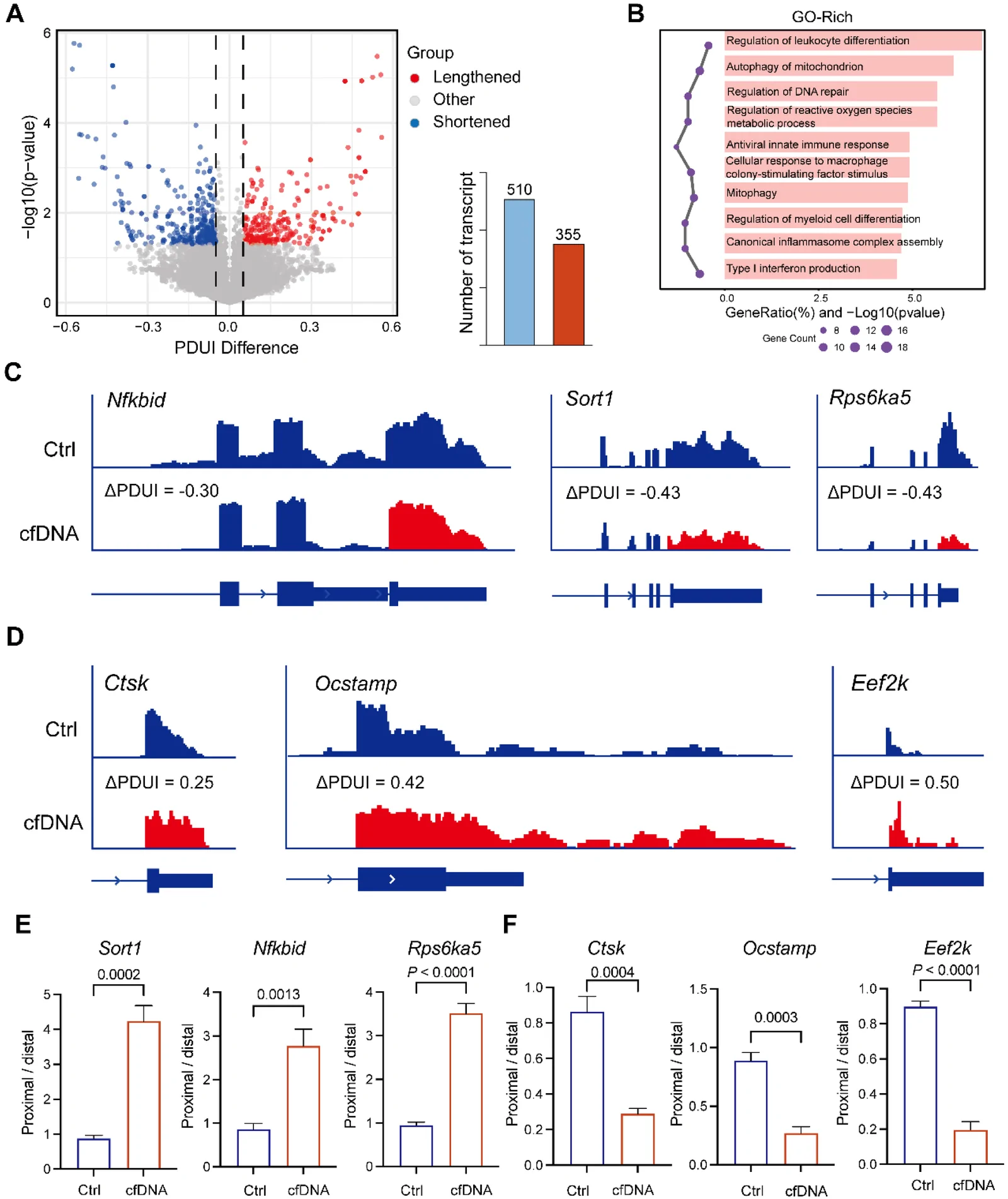
**APA analysis of RAW264.7 cells after cfDNA stimulation.** (A) DaPars2-derived volcano plot depicting globally shortened and lengthened 3′UTRs post-cfDNA stimulation. (B) GO enrichment analysis of genes undergoing 3′UTR lengthening. (C) IGV tracks visualizing the 3′UTR read coverage of representative M1-associated pro-inflammatory genes (*Nfkbid, Sort1, Rps6ka5*) featuring proximal poly(A) site usage (shortened 3′UTR) and (D) M2-associated genes (*Ctsk, Ocstamp, Eef2k*) featuring distal polyA site usage (lengthened 3′UTR). (E) RT-qPCR quantification of proximal-to-distal isoform ratios validating the APA shifts in M1-related and (F) M2-related genes upon cfDNA treatment. n = 3 independent biological replicates. Statistical significance was determined using a paired *t*-test.

To rigorously validate this APA-driven polarization hypothesis at single-gene resolution, we visualized the RNA-seq read-coverage tracks of key macrophage functional genes using the Integrative Genomics Viewer (IGV). The tracks demonstrated that a series of classical pro-inflammatory and osteoclastogenic (M1-related) driver genes—such as *Sort1* (ΔPDUI = −0.43), *Rps6ka5* (ΔPDUI = −0.43) and *Nfkbid* (ΔPDUI = −0.30)—exhibited a pronounced preference for proximal polyadenylation site (PAS) cleavage upon cfDNA stimulation, resulting in markedly shortened 3′UTR (Fig. 2C). Conversely, key genes vital for M2 polarization and immune homeostasis, such as *Eef2k* (ΔPDUI = 0.50), *Ctsk* (ΔPDUI = 0.25) and *Ocstamp* (ΔPDUI = 0.42), predominantly utilized distal poly(A) sites, yielding significantly lengthened 3′UTR (Fig. 2D).

To experimentally verify these bioinformatic findings in vitro, we designed specific primers targeting the proximal and distal 3′UTR regions of these pivotal genes and quantified the isoform ratios via quantitative real-time PCR (RT-qPCR). As anticipated, for M1-associated genes (e.g., *Sort1*), the ratio of proximal-to-distal transcripts surged drastically in the cfDNA group (Fig. 2E). This confirmed a massive transcriptomic shift toward short-3′UTR isoforms, thereby unleashing the translational potential of these pro-inflammatory genes. In stark contrast, the proximal/distal ratio for M2-associated genes (e.g., *Ctsk*) plummeted following cfDNA stimulation (Fig. 2F), affirming the dominance of long-3′UTR isoforms that effectively silence the macrophage anti-inflammatory program.

### 2.3 Synthesis and characterization of the DA-APAT nanoplatform

Thoroughly depleting cfDNA from the inflammatory microenvironment has emerged as a core intervention strategy to halt periodontitis progression. Based on the pathogenic mechanism and diagnostic potential of cfDNA, we logically designed and engineered a dual-action nanotheranostic platform, termed Dual-Action APA Theranostics (DA-APAT). As illustrated in the synthetic scheme (Fig. 3A), a hard-templating strategy was initially employed to grow a CeO_2_ shell in situ on silica (SiO_2_) nanosphere templates. Subsequent NaOH etching removed the SiO_2_ core, yielding hollow mesoporous cerium oxide (HMCeO_2_) nanoparticles. HMCeO_2_ not only possesses intrinsic antioxidant nanozyme activity—which is crucial for alleviating oxidative stress in the inflammatory microenvironment—but its spacious hollow cavity also provides an ideal spatially confined environment for loading deoxyribonuclease I (DNase I). Finally, polyethylenimine (PEI) was electrostatically coated onto the outermost layer to construct the complete DA-APAT. The highly polycationic PEI shell is engineered to precisely and efficiently capture negatively charged cfDNA from the microenvironment via electrostatic interactions, endowing the platform with a potential capacity for quantitative cfDNA diagnostics. Following capture, the cfDNA is shuttled into the mesoporous channels for thorough enzymatic degradation by the internally loaded DNase I. This highly efficient "Capture-and-Cleave" cascade is fundamentally designed to rescue the globally disrupted APA landscape.

**Fig. 3:**
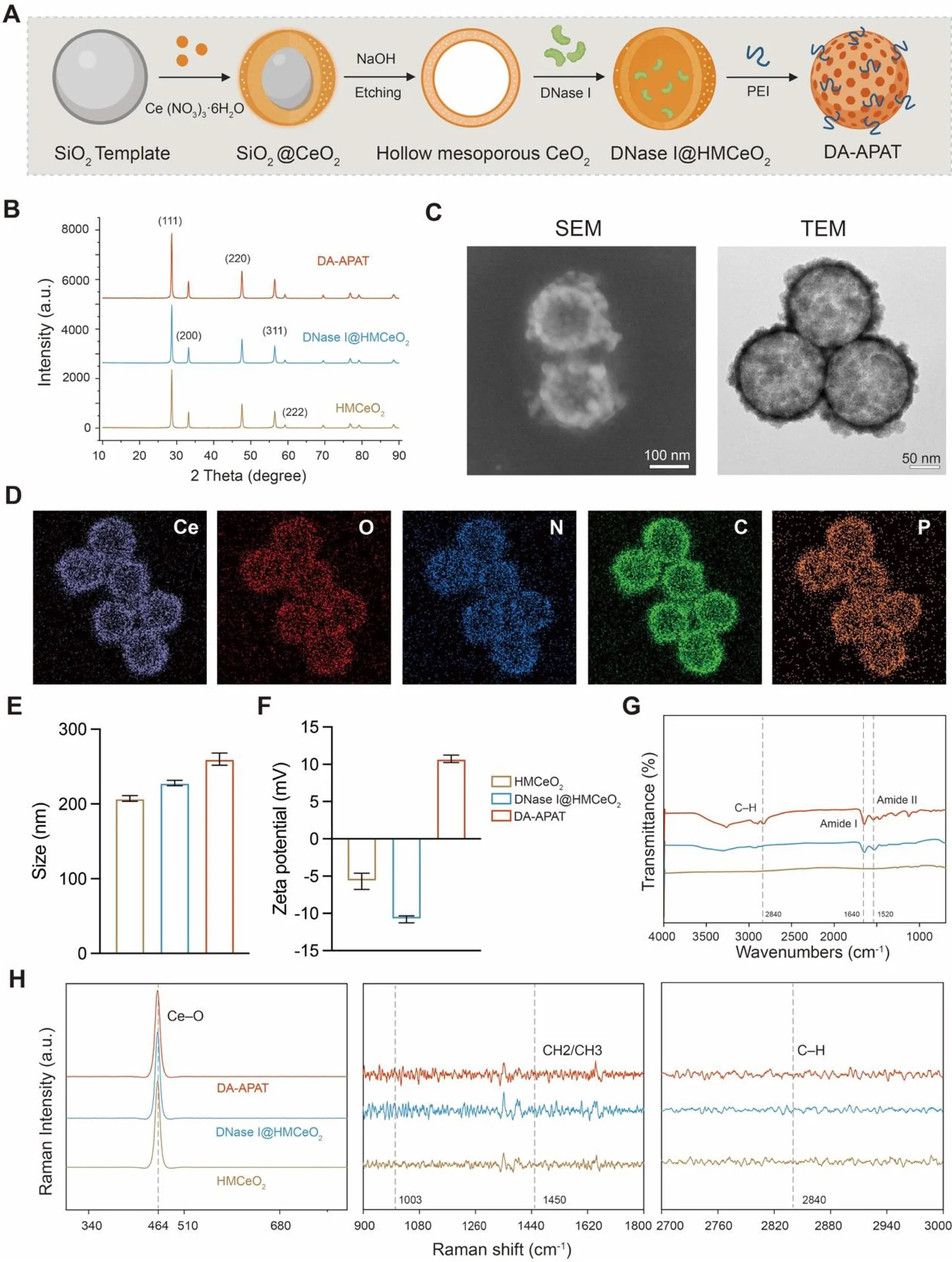
**Design, synthesis, and physicochemical characterization of the DA-APAT nanoplatform.** (A) Schematic illustration of the synthesis of DA-APAT. (B) XRD patterns of HMCeO_2_ during sequential assembly. (C) Representative SEM and TEM images of DA-APAT. (D) EDS elemental mapping of Ce, O, C, N, and P. (E) Hydrodynamic size distributions measured by DLS during DA-APAT assembly. (F) Zeta potentials of HMCeO_2_, DNase I@HMCeO_2_, and DA-APAT. (G) FTIR spectra and (H) Raman spectra of HMCeO_2_, DNase I@HMCeO_2_, and DA-APAT.

To verify the successful construction of this hierarchical nanoplatform, a series of rigorous physicochemical characterizations were performed. First, X-ray diffraction (XRD) analysis confirmed the crystal structures. HMCeO_2_, DNase I@HMCeO_2_, and the final DA-APAT all exhibited characteristic diffraction peaks at the (111), (200), (220), (311), and (222) planes, perfectly matching the classical cubic fluorite structure of CeO_2_ (Fig. 3B). This indicates that the multistep assembly and protein loading processes did not compromise the cerium oxide crystalline framework. Morphologically, scanning electron microscopy (SEM) and transmission electron microscopy (TEM) images explicitly revealed the uniform spherical morphology and the well-defined "hollow mesoporous" structure of DA-APAT (Fig. 3C). This unique architecture provides ample payload capacity for the macromolecular DNase I while ensuring unobstructed mesoporous channels for cfDNA influx and degraded fragment efflux. Furthermore, energy-dispersive X-ray spectroscopy (EDS) elemental mapping clearly demonstrated the co-localization of Ce, O, C, N, and P. While Ce and O originated from the inorganic metal shell, the uniform distribution of C, N, and P provided compelling evidence for the successful integration of PEI and DNase I onto the nanoparticles (Fig. 3D).

From a hydrodynamic perspective, dynamic light scattering (DLS) and surface Zeta potential analyses dynamically tracked the layer-by-layer assembly trajectory of DA-APAT. The hydrodynamic diameter exhibited a stepwise increase during the sequential loading: from 200 nm for the bare HMCeO_2_, to 220 nm post-DNase I loading, and finally stabilizing at approximately 260 nm after PEI coating (Fig. 3E). Concurrently, the Zeta potential underwent a hallmark reversal. Bare HMCeO_2_ was slightly electronegative (−5 mV); upon loading the negatively charged DNase I protein, the potential dropped to −10 mV. However, successful outer wrapping with the cationic polymer PEI abruptly inverted the surface charge of the final DA-APAT to +10 mV (Fig. 3F). Notably, both the particle size and Zeta potential of DA-APAT remained robustly stable over a one-week storage period (Fig. S3), corroborating excellent structural stability.

Chemically, Fourier-transform infrared (FTIR) spectroscopy displayed a relatively flat baseline for HMCeO_2_ in the mid-infrared region. Following DNase I encapsulation, two prominent characteristic peaks emerged at approximately 1640 cm^−1^ and 1520 cm^−1^, corresponding to the amide I (C=O stretching) and amide II (N-H bending) bands of the protein, respectively. Additionally, a strong C-H stretching vibration peak at 2840 cm^−1^ confirmed the successful PEI modification (Fig. 3G). Raman spectroscopy further validated these findings: alongside the characteristic Ce-O symmetric stretching vibration at 464 cm^−1^, the significantly enhanced signals at 1450 cm^−1^ (CH2/CH3 deformation) and 2840 cm^−1^ (C-H stretching) strongly verified the successful synthesis of DA-APAT (Fig. 3H). Crucial to its therapeutic application, the DA-APAT nanoplatform exhibited an excellent DNase I encapsulation efficiency of 70.15% (Fig. S4B). To further validate the stability of the loaded enzyme, we tracked the in vitro release kinetics over a 14-day period at 4 °C (Fig. S4D). Both the intermediate DNase I@HMCeO_2_ and the fully assembled DA-APAT displayed a sustained enzyme release profile. Quantitatively, thermogravimetric analysis (TGA) demonstrated that pure HMCeO_2_ exhibited negligible weight loss (∼1.77%), reflecting high thermal stability. After DNase I loading, the total weight loss reached 4.18%, precisely corresponding to a 2.41 wt% enzyme loading capacity. Following PEI modification, the weight loss dramatically increased to 17.53%, indicating a net PEI grafting ratio of 13.35 wt% (Fig. S5).

To dissect the synergistic 'capture-and-degrade' mechanism of our nanoplatform and to clarify the indispensable roles of its individual components, we quantified the residual cfDNA in both the coincubation supernatant and within the materials themselves. As shown in Fig. S6, when incubated with bare HMCeO_2_ or DNase I@HMCeO_2_, high levels of cfDNA remained in the supernatant, indicating an inferior capturing ability without the polycationic PEI shell. In stark contrast, DA-APAT significantly depleted the cfDNA from the supernatant (approx. 17.5 ng/mL). Crucially, the quantification of cfDNA retained on/within the nanoparticles revealed that DA-APAT possessed the lowest residual cfDNA content (approx. 3.5 ng/mL) compared to HMCeO_2_ (approx. 32.1 ng/mL) and DNase I@HMCeO_2_ (approx. 26.2 ng/mL). This compellingly demonstrates that DA-APAT does not merely sequester cfDNA via physical adsorption, but rapidly and thoroughly degrades it via the entrapped DNase I, successfully preventing the detrimental accumulation of intact immunostimulatory DNA on the material surface.

Collectively, these comprehensive structural and compositional characterizations definitively confirm the successful fabrication of the logically assembled, physicochemically stable DA-APAT theranostic system.

### 2.4 DA-APAT is helpful for the early diagnosis and subsequent treatment of periodontitis

Prior to advancing to biological evaluations, the cytotoxicity of the nanomaterials was assessed using the CCK-8 assay. As depicted in Fig. 4A, even when the concentrations of HMCeO_2_ and the fully assembled DA-APAT were increased up to 20 μg/mL, cellular viability remained robustly above 78%, establishing a solid safety profile for subsequent biomedical applications. Importantly, to investigate how DA-APAT modulates the intracellular trafficking of cfDNA, we performed confocal microscopy tracking (Fig. S7). Free cfDNA was primarily confined within the lysosomes. Notably, the HMCeO_2_-PEI group facilitated robust cellular internalization via the “effect of PEI”. The abundant protonatable amine groups of PEI provide substantial buffering capacity in the acidic endosomal environment, which can facilitate endosomal escape through proton buffering, osmotic effects, and membrane destabilization [40], [41]. In contrast, in the DA-APAT group, although fragmented red fluorescence signals were observed in the cytoplasm due to the dye-conjugated nucleotides, the core DNase I rapidly cleaved the intact cfDNA into short oligonucleotides, neutralizing their immunostimulatory potential.

**Fig. 4:**
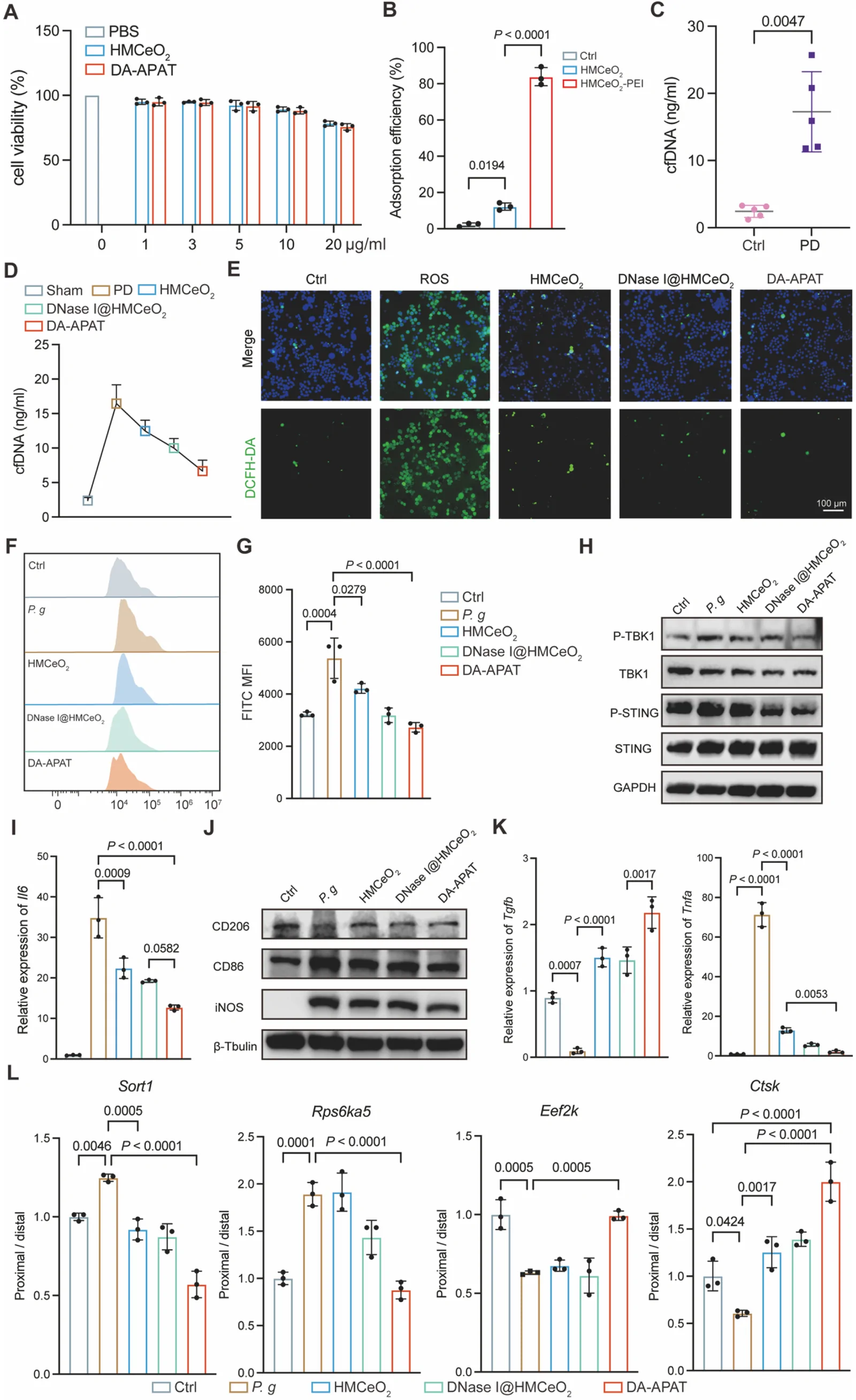
**DA-APAT mediates cfDNA clearance, ROS scavenging, and macrophage repolarization in vitro.** (A) CCK-8 assay of nanomaterial cytocompatibility. (B) In vitro cfDNA adsorption efficiency of HMCeO_2_-PEI measured by PicoGreen assay. (C) Quantification of cfDNA adsorbed by HMCeO_2_-PEI in saliva from Ctrl and PD mice (n = 5). (D) Residual cfDNA levels in mouse saliva. (E) Representative confocal images of DCFH-DA (green) and DAPI (blue) staining. (F) Flow cytometry analysis and (G) quantification of FITC mean fluorescence intensity (MFI). (H) Western blot analysis of STING, p-STING, TBK1, and p-TBK1. (I) RT-qPCR analysis of Il6 expression. (J) Western blot analysis of CD86, iNOS, and CD206. (K) RT-qPCR analysis of *Tgfb* and *Tnfa* expression. (L) RT-qPCR quantification of proximal-to-distal 3′UTR isoform ratios. Data are presented as mean ± SD from at least three independent biological experiments unless otherwise indicated. Statistical significance was determined by one-way ANOVA followed by Tukey's multiple-comparisons test. (For interpretation of the references to color in this figure legend, the reader is referred to the Web version of this article.)

To validate the function of this system, we first formulated an enzyme-free precursor (HMCeO_2_-PEI) and directly quantified its cfDNA adsorption capacity in a simulated microenvironment. The PicoGreen assay revealed that HMCeO_2_-PEI achieved an exceptional cfDNA adsorption efficiency exceeding 80% (Fig. 4B). Advancing this concept towards clinical translatability, we collected saliva samples from both healthy and P. g-induced periodontitis mice for nanoparticle-based detection. A standard curve confirmed the high accuracy of the concentration measurements (R^2^ = 0.999, Fig. S8). Scatter plots demonstrated that HMCeO_2_-PEI sensitively captured and quantified the abundant cfDNA released into the saliva of diseased mice (Fig. 4C). Having verified the in vitro capture capability, we next evaluated the complete cfDNA degradation efficacy of DA-APAT in vivo. The in vivo kinetic profiles clearly illustrated that local cfDNA concentrations remained abnormally high in the periodontal lesions of diseased mice. Treatment with bare HMCeO_2_ barely altered this trajectory, while DNase I@HMCeO_2_ showed marginal effects due to the lack of active targeting and capture capabilities. In stark contrast, the complete DA-APAT exhibited remarkable superiority: driven by the "PEI capture → DNase I cleavage" cascade, it plummeted the local cfDNA concentration almost back to baseline physiological levels (Fig. 4D). In conclusion, We first assessed the diagnostic potential of the PEI shell (HMCeO_2_-PEI) by quantifying salivary cfDNA levels, confirming their elevation in periodontitis (Fig. 4C). We then evaluated the therapeutic efficacy of the full DA-APAT platform in clearing cfDNA in vivo (Fig. 4D).

It is noteworthy that previously reported cfDNA-scavenging materials were predominantly deployed as late-stage salvage therapies for systemic hyperinflammatory diseases (e.g., sepsis or rheumatoid arthritis) [42], [43]. However, the clinical management of periodontitis presents entirely distinct stomatological demands. The periodontal field urgently requires strategies capable of early asymptomatic screening, meticulous monitoring of tissue recovery following basic mechanical treatments (e.g., scaling and root planing), and precise optimization of the immune microenvironment prior to dental implant placements. DA-APAT platform perfectly bridges this gap. Because of its highly sensitive salivary cfDNA diagnostic capability, it not only serves as a robust therapeutic scavenger during peak inflammatory bursts but also enables fine-tuned, dynamically matched interventions during early onset or post-treatment recovery phases. Thus, the choice of the orthotopic periodontitis model herein is not merely a proof-of-concept; it highlights a highly specialized clinical scenario where this homologous "diagnostic-guided clearance" design is uniquely irreplaceable.

Given that the core of DA-APAT is constructed from HMCeO_2_, which possesses intrinsic antioxidant nanozyme properties, we evaluated its ROS-quenching capability in RAW 264.7 macrophages using a DCFH-DA fluorescent probe. Confocal microscopy revealed intense, diffuse green fluorescence within cells under severe oxidative stress (ROS-positive control). Strikingly, treatment with DA-APAT drastically abolished this fluorescent signal (Fig. 4E). The levels of ROS produced by *P. g*-stimulated cells were measured by flow cytometry. Both the distinctive leftward shift in flow cytometric histograms (Fig. 4F) and the quantitative mean fluorescence intensity (MFI) analysis (Fig. 4G) confirmed that DA-APAT profoundly scavenges aberrantly accumulated intracellular ROS, restoring them to basal levels.

The canonical downstream signaling axis responsible for cytosolic cfDNA sensing is the cGAS-STING pathway [19]. Accordingly, we investigated the activation status of this crucial cascade. Western blot analysis revealed that *P. g* infection triggered massive phosphorylation and activation of STING and TBK1 (p-STING, p-TBK1). However, benefiting from the highly efficient cfDNA clearance by DA-APAT, the activation of the intracellular STING signaling axis was robustly abrogated, evidenced by significantly diminished phosphorylation levels (Fig. 4H). In tandem with STING pathway silencing, the mRNA transcription of the downstream core pro-inflammatory cytokine IL-6 was profoundly suppressed (Fig. 4I). Most crucially, validating our central hypothesis, DA-APAT effectively orchestrated a reversal of macrophage polarization. While the *P. g*-infected group exhibited classical M1 phenotypic features (hyper-expression of the pro-inflammatory markers CD86 and iNOS, with a near-complete absence of the anti-inflammatory marker CD206), DA-APAT treatment not only severely downregulated CD86 and iNOS but also triggered substantial expression of the tissue-repairing/anti-inflammatory M2 marker CD206 (Fig. 4J). This protein-level macrophage repolarization trend was further corroborated by cytokine mRNA quantification: in the DA-APAT-treated group, the mRNA levels of M2-associated anti-inflammatory cytokines (*Tgfb1* and *Il4*) surged significantly, whereas prototypical M1 pro-inflammatory cytokines (*Tnf* and *Il1b*) were potently suppressed (Fig. 4K, Fig. S9).

To prove the indispensable role of DNase I, we compared the anti-inflammatory efficacy of DA-APAT with the enzyme-free HMCeO_2_-PEI control (Fig. S10). Immunofluorescence analysis revealed that DA-APAT profoundly promoted M2 polarization (ARG1^+^) and suppressed M1 polarization (iNOS^+^). In stark contrast, HMCeO_2_-PEI exhibited negligible therapeutic effects. Consistently, RT-qPCR analyses demonstrated that DA-APAT significantly downregulated pro-inflammatory cytokines (*Il6, Il1b, Tnfa*) and upregulated anti-inflammatory markers (*Il4, Tgfb*), whereas HMCeO_2_-PEI failed to rescue the inflammatory phenotype (Fig. S11), highlighting that physical adsorption alone is insufficient and enzymatic degradation is imperative.

Furthermore, to definitively exclude the potential interference of LPS and other PAMPs present in live *P. gingivalis*, we evaluated the specific therapeutic efficacy of DA-APAT in a pure cfDNA-induced macrophage model. Consistent with the bacterial infection model, RT-qPCR analyses confirmed that DA-APAT robustly downregulated cfDNA-triggered pro-inflammatory genes (*Il6, Il1b, Tnfa*) while significantly upregulating anti-inflammatory markers (*Il4, Tgfb*) (Fig. S12). More importantly, Western blot analysis revealed that pure cfDNA stimulation directly triggered robust M1 polarization (upregulation of iNOS and CD86) and strongly activated the precise target pathway (marked elevation of total STING and p-STING). Only the comprehensive DA-APAT treatment successfully and comprehensively abrogated the expression of these pathway proteins (Fig. S13), unequivocally confirming its precise interception of the cfDNA-cGAS-STING pathogenic axis.

To definitively close the mechanistic loop and ascertain whether this robust immunomodulatory efficacy genuinely stems from the correction of post-transcriptional rewiring, we finally quantified the targeted APA events in *P. g*-infected macrophages across the various treatment groups. Utilizing RT-qPCR to measure the proximal-to-distal 3′UTR isoform ratios, we observed that *P. g* infection sharply increased the proximal/distal ratio of M1-associated drivers (e.g., *Sort1* and *Rps6ka5*), propelling their hyper-translation, while suppressing M2/tissue-homeostatic genes (e.g., *Eef2k* and *Ctsk*) by drastically lowering their proximal/distal ratios (Fig. 4L). Crucially, the intervention strategies yielded highly distinct outcomes that perfectly corroborated our rational nanoplatform design. Bare HMCeO_2_ intervention showed limited effects on rescuing the APA imbalance, as it solely scavenges ROS without addressing the upstream cfDNA trigger. The DNase I@HMCeO_2_ intermediate displayed a modest restorative trend; however, lacking the critical PEI "capture net", its passive, unguided enzymatic degradation was insufficient to fully reverse the severe transcriptomic dysregulation within the aggressive *P. g*-induced inflammatory milieu. In stark contrast, the fully assembled DA-APAT platform decisively and completely remodeled the APA landscape back to near or even exceed the baseline levels.

### 2.5 DA-APAT rescues alveolar bone loss and facilitates periodontal tissue restoration

To determine whether DA-APAT could exert its robust immunomodulatory efficacy within a complex in vivo pathological microenvironment, we established a classical experimental periodontitis (PD) murine model and evaluated the in vivo therapeutic outcomes. Three-dimensional Micro-CT reconstructions (Fig. 5A) and corresponding sagittal tomograms (Fig. 5B) vividly delineated the skeletal morphology across all groups. In the untreated PD group, the distance from the cementoenamel junction (CEJ) to the alveolar bone crest (ABC) increased drastically, accompanied by severe alveolar bone fenestration and massive horizontal/vertical resorption. Remarkably, mice treated with DA-APAT maintained an almost intact alveolar bone contour with dense and continuous bone architecture (Fig. S14). Bone histomorphometric analysis provided unequivocal quantitative support: the DA-APAT-treated group exhibited highly significant improvements in bone volume fraction (BV/TV), trabecular number (Tb.N), and trabecular thickness (Tb.Th), alongside a substantial reduction in trabecular separation (Tb.Sp) (Fig. 5C).

**Fig. 5:**
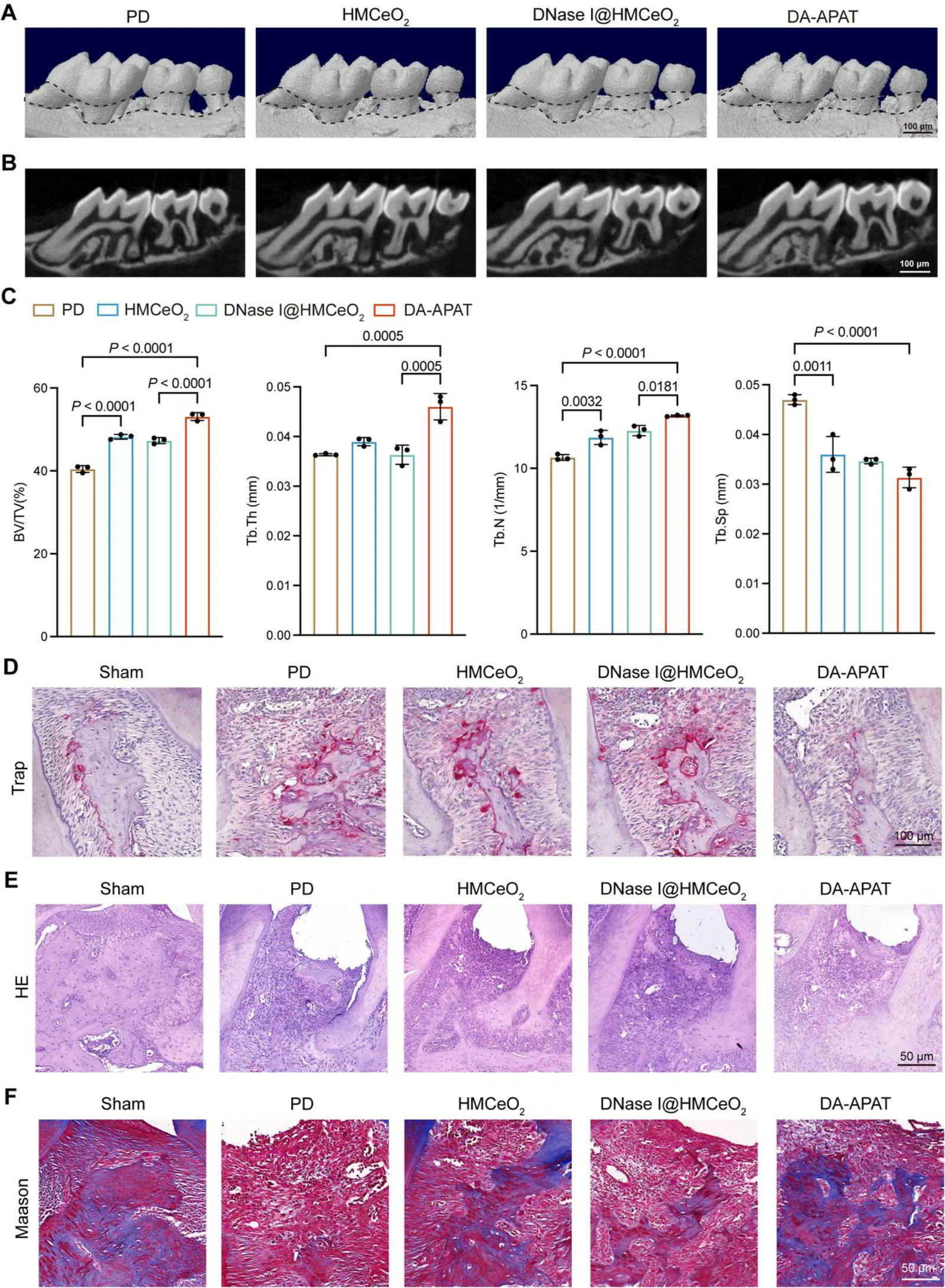
**DA-APAT exerts exceptional therapeutic efficacy in halting alveolar bone loss and driving periodontal tissue restoration.** (A) Representative 3D Micro-CT reconstructed images and (B) sagittal tomograms of maxillary molars displaying alveolar bone architectures across different groups. (C) Quantitative bone histomorphometric analyses including BV/TV, Tb.N, Tb.Th, and Tb.Sp. (D) TRAP staining images of periodontal sections. (E) H&E staining illustrating the inflammatory cell infiltration. (F) Representative images of masson's trichrome staining demonstrating the condition of collagen fibers. Data are presented as mean ± SD from at least three independent biological experiments unless otherwise indicated. Statistical significance was determined by one-way ANOVA followed by Tukey's multiple-comparisons test.

Histological assessments further unveiled the cellular and matrix-level mechanisms underlying this profound osteoprotection. Tartrate-resistant acid phosphatase (TRAP) staining revealed an massive accumulation of deeply stained, multinucleated osteoclasts lining the bone margins in the PD group. Conversely, DA-APAT intervention thoroughly suppressed osteoclast differentiation and resorptive activity, an effect functionally linked to the successful microenvironmental shift toward the M2 anti-inflammatory phenotype (Fig. 5D). Hematoxylin and eosin (H&E) staining corroborated that DA-APAT therapy effectively eradicated local inflammatory cell infiltration and restored the highly ordered arrangement of the periodontal ligament (PDL) (Fig. 5E). Concurrently, Masson's trichrome staining demonstrated that newly deposited collagen fibers in the treated periodontal tissues were dense and highly organized, manifesting a robust capacity for soft tissue repair and regeneration (Fig. 5F).

To advance the clinical translational potential of this nanotheranostic platform, we systematically evaluated its in vivo biocompatibility. H&E staining of major vital organs (heart, liver, spleen, lungs, and kidneys) revealed that following a complete therapeutic regimen, DA-APAT induced no discernible histological lesions or systemic toxicity (Fig. S8). Collectively, these findings establish that DA-APAT combines an excellent safety profile with a cutting-edge cfDNA-targeted depletion mechanism, culminating in exceptional periodontal tissue restoration.

### 2.6 DA-APAT silences in vivo DNA-sensing pathways and repolarizes macrophages

To definitively map the in vivo therapeutic action pathways of DA-APAT, we investigated the activation status of TLR9 and STING within the periodontal tissues. Immunofluorescence (IF) confocal imaging demonstrated that, compared to the Sham group, the expression of TLR9 was hyperactivated in the periodontium of PD mice. However, following localized DA-APAT intervention—which efficiently degraded the ambient cfDNA—the mean fluorescence intensity (MFI) of TLR9 plummeted to near-healthy baseline levels (Fig. 6A). This "root-clearing" efficacy was highly consistent with the immunohistochemical (IHC) staining of the STING pathway: statistical analysis confirmed that DA-APAT intervention drastically reduced the STING-positive expression area adjacent to the alveolar bone (Fig. 6B). These data indicate that by eradicating the upstream cfDNA trigger, DA-APAT successfully short-circuits the central hubs of inflammatory amplification at the in vivo level.

**Fig. 6:**
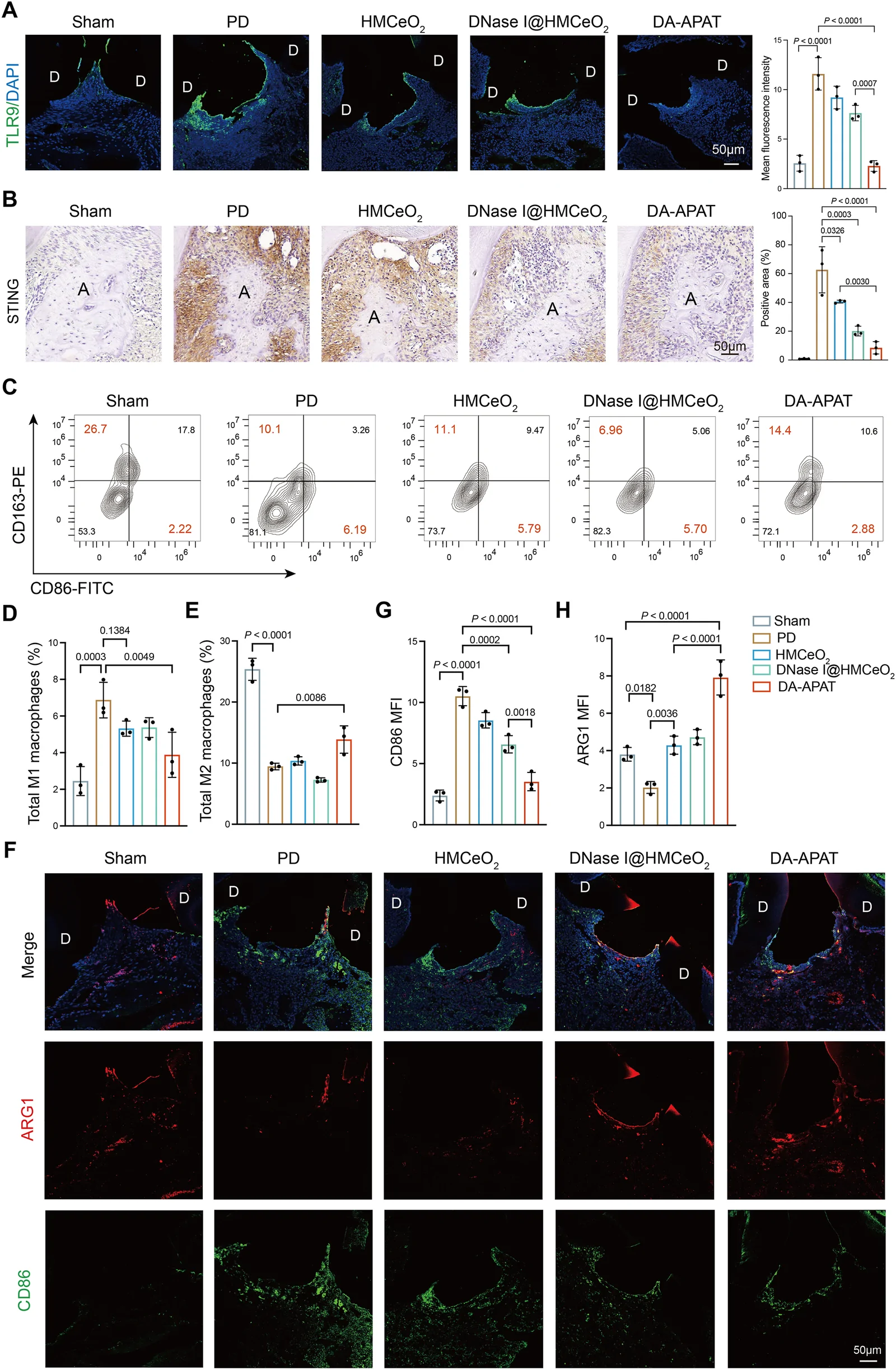
**DA-APAT silence DNA-sensing hubs and reversing macrophage polarity.** (A) IF confocal imaging and corresponding MFI quantification of TLR9 expression in periodontal tissues. D, dental. (B) IHC staining and positive area percentage of STING in the alveolar bone vicinity. A, alveolar bone. (C) Flow cytometry pseudocolor plots identifying macrophage within the gingival microenvironment. (D) Quantitative statistical graphs of flow cytometry showing the CD86^+^ M1 subsets and (E) CD163^+^ M2 subsets. (F) Co-immunofluorescence staining of CD86 (red) and ARG1 (green) in periodontal sections. (G) Quantitative fluorescence intensities of CD86 and (H) ARG1 in situ. Statistical significance was determined using a one-way analysis of variance (ANOVA) followed by Tukey's multiple comparisons test. (For interpretation of the references to color in this figure legend, the reader is referred to the Web version of this article.)

Consequent to the silencing of these DNA-sensing pathways, profound polarization shifts occur within the immune microenvironment. To profile this, we isolated immune cells from murine gingival tissues and subjected them to flow cytometry analysis. Following a standard gating strategy for macrophage subsets (Fig. S9), we observed that the destructive CD86^+^ M1 macrophages absolutely dominated the PD microenvironment, whereas the tissue-repairing CD163^+^ M2 macrophages were severely depleted. Strikingly, DA-APAT therapy forcefully rectified this immune dysbiosis (Fig. 6C). Quantitative analysis revealed a massive contraction of the CD86^+^ M1 population, coupled with an explosive expansion of the CD163^+^ M2 subset (Fig. 6D and E). To visually corroborate this phenomenon in situ, we performed co-immunofluorescence staining for CD86 and Arginase-1 (ARG1, a canonical M2 marker) on periodontal sections (Fig. 6F). As verified by fluorescence quantification, the DA-APAT-treated group exhibited a marked decline in CD86 MFI and a significant surge in ARG1 expression (Fig. 6G and H). Together, these in vivo results robustly demonstrate that by intercepting cfDNA, DA-APAT not only severs the TLR9/STING-driven inflammatory cascade but, more importantly, reprograms macrophage polarization. This restores the intrinsic tissue-repair functions of M2 macrophages, ultimately driving profound periodontal repair.

## 3 Discussion and conclusion

The intricate host-pathogen battleground in periodontitis leads to massive cell death and the extracellular accumulation of cfDNA. While previously recognized merely as metabolic debris, our study firmly establishes cfDNA as a fundamental pathogenic instigator. Our previous work uncovered that *P. gingivalis* infection severely disrupts post-transcriptional homeostasis, compelling pro-inflammatory macrophages to adopt shortened 3′UTR [32]. However, the exact extracellular mediator driving this phenomenon remained elusive. By mapping the comparative transcriptomic landscape, we pinpointed that it is the accumulated cfDNA—following pathogenic infection—that persistently hijacks the RNA processing machinery. Specifically, cfDNA disrupts the global alternative polyadenylation (APA) equilibrium, forcing pro-inflammatory transcripts into translationally hyperactive short-3′UTR isoforms while subjecting anti-inflammatory M2 programs to post-transcriptional silencing via 3′UTR lengthening. Biologically, *Sort1* promotes inflammatory responses via the Wnt/β-catenin pathway and acts as a high-affinity receptor for IL-6 and IFN-γ, decisively driving M1 polarization [44], [45]. *Rps6ka5* encodes the MSK1 protein, which orchestrates macrophage inflammation via TLR signaling [46], while *Nfkbid* modulates the NF-κB transcriptional profile. *Ocstamp* was initially identified as a transmembrane protein involved in osteoclast fusion; however, recent studies have demonstrated that it is also expressed in macrophages and participates in the regulation of inflammatory responses. Under M2 polarization conditions, *Ocstamp* expression is markedly upregulated, thereby contributing to anti-inflammatory activity and tissue repair [47]. CTSK, a cysteine protease highly expressed in osteoclasts, has likewise been increasingly recognized for its roles in inflammation regulation and macrophage polarization [48]. In our study, we found that stimulation with cfDNA led to a significant extension of the 3′UTR of M2-associated genes, which was accompanied by suppression of their expression. This cfDNA-driven APA rewiring provides a crucial mechanistic explanation for why immune cells become permanently locked into a destructive phenotype during chronic periodontitis, despite conventional antimicrobial treatments.

To translate this biological discovery into an actionable intervention, we rationally engineered the DA-APAT nanoplatform. Unlike passive ROS scavengers or naked nucleases that suffer from rapid clearance and lack of targeting, DA-APAT was constructed with a highly specific "Capture-and-Cleave" synergy. As evidenced by our in vitro APA rescue experiments (Fig. 4L and M), bare HMCeO_2_ failed to correct the transcriptomic dysregulation due to its inability to clear upstream cfDNA. Similarly, the DNase I@HMCeO_2_ intermediate yielded insufficient recovery, as passive enzyme diffusion struggles to efficiently neutralize abundant ambient DAMPs. It was only the integration of the polycationic PEI shell—acting as an electrostatic "net"—that successfully enriched the microenvironmental cfDNA and shuttled it into the mesoporous cavity for thorough enzymatic degradation. This precisely engineered structural complementarity underscores the necessity of advanced delivery biomaterials in executing targeted epigenetic/RNA-regulatory rescue.

Furthermore, while the current platform does not aim to replace established clinical diagnostics with absolute quantitative thresholds, its extraordinary ability to enrich ultra-trace cfDNA offers a distinct advantage for longitudinal disease monitoring. In periodontitis, the fluctuation of salivary cfDNA concentration is not merely a binary indicator, but rather a dynamic reflection of real-time inflammatory activity, immune cell death (e.g., NETosis), and the severity of alveolar bone destruction. By utilizing the polycationic PEI shell as an enrichment strategy, this platform paves a promising 'liquid biopsy' avenue for non-invasively tracking disease progression, evaluating therapeutic efficacy, and predicting relapse in personalized periodontal care. Despite the profound therapeutic efficacy demonstrated by DA-APAT, this study has certain limitations that warrant future investigation. While our transcriptomic and in vitro data strongly implicate the cfDNA-cGAS-STING axis in driving the pathological APA landscape and macrophage M1 polarization, the current study lacks validation via absolute genetic deletion. Future research utilizing macrophage-specific conditional knockout models (e.g., cGAS^flox/flox^ or Sting1^flox/flox^ mice) will be essential to definitively establish the exclusive causal reliance of the APA remodeling on this specific cytosolic DNA-sensing pathway. From a translational perspective, it is important to note that while local gingival injection was employed in this study to ensure precise and standardized dosing in the murine model, this invasive delivery method is less ideal for human clinical practice. For future clinical applications, the versatile DA-APAT nanoparticles can be seamlessly incorporated into advanced, non-invasive delivery vehicles tailored for the periodontal pocket. For instance, formulating DA-APAT into thermo-sensitive in situ hydrogels (e.g., Poloxamer 407/Pluronic F-127) would allow the formulation to be administered as a flowable liquid that instantly gels upon entering the warm periodontal pocket [49], [50], [51], ensuring prolonged retention and sustained release of DNase I. Alternatively, incorporation into dental ointments or prolonged-release polymer chips (similar to clinical chlorhexidine chips) could provide localized, continuous "capture-and-degrade" therapeutic action without the need for invasive injections, thereby significantly enhancing patient compliance and clinical applicability.

Ultimately, by actively scavenging pathogenic cfDNA and synergistically quenching ROS, DA-APAT successfully short-circuits the canonical intracellular DNA-sensing hubs (cGAS-STING and TLR9). The silencing of these pathways rectifies the upstream signaling cascade, effectively reversing the APA dysregulation and unlocking the tissue-repairing potential of M2 macrophages. The robust alveolar bone preservation and organized collagen repair observed in vivo highlight the immense potential of modifying the post-transcriptional immune microenvironment for tissue engineering applications. This work not only provides profound molecular insights into cfDNA-mediated immune dysregulation but also establishes a highly translatable, diagnostic-guided precision nanomedicine modality for periodontitis and other cfDNA-associated inflammatory diseases.

## 4 Materials and methods

### 4.1 Synthesis of hollow mesoporous cerium oxide (HMCeO_2_) nanoparticles

Uniform silica (SiO_2_) nanospheres were first synthesized as hard templates using a modified Stöber method. Next, to coat the CeO_2_ shell, 100 mg of SiO_2_ templates were dispersed in 100 mL of deionized (DI) water containing 400 mg of cerium nitrate hexahydrate (Ce(NO_3_)_3_·6H_2_O). After adding 400 mg of hexamethylenetetramine, the mixture was stirred at 90 °C for 6 h. The resulting core-shell SiO_2_@CeO_2_ nanoparticles were collected by centrifugation and calcined at 500 °C for 3 h. Finally, the SiO_2_ cores were selectively etched away using 2 M NaOH solution at 50 °C for 12 h, yielding HMCeO_2_ nanoparticles.

### 4.2 Fabrication of the DA-APAT nanoplatform

The assembly of the Dual-Action APA Theranostics (DA-APAT) was achieved through a sequential, layer-by-layer loading and electrostatic coating strategy.

To prepare DNase I-loaded HMCeO_2_-PEI nanoparticles, 10 mg of the synthesized HMCeO_2_ nanoparticles was uniformly dispersed in 10 ml of DI water (1.0 mg/ml) via brief sonication. Subsequently, 2 mg of DNase I (Sigma-Aldrich, USA, Cat. 11284932001) was added to the suspension to obtain a final DNase I concentration of 0.2 mg/ml. The mixture was incubated under continuous gentle stirring at 4 °C overnight to allow for maximum pore diffusion and protein entrapment. Following incubation, the DNase I@HMCeO_2_ intermediate was collected by centrifugation (10,000 rpm for 10 min) and washed twice with DI water to remove any unbound enzyme.

For PEI modification, the purified DNase I@HMCeO_2_ nanoparticles were redispersed in 10 ml of DI water. Branched polyethylenimine (PEI, MW = 25 kDa, Polysciences, USA) was then added at a concentration of 0.5 mg/mL (5 mg in 10 ml). The electrostatic self-assembly process was allowed to proceed overnight at 4 °C under gentle rotation. Finally, the fully assembled DA-APAT nanoparticles were harvested by centrifugation, washed thoroughly with DI water to completely remove unreacted free PEI, and ultimately resuspended in sterile PBS or stored at 4 °C for subsequent in vitro and in vivo evaluations.

### 4.3 Material characterizations

The morphological architectures were visualized using a scanning electron microscope (SEM, Tescan CLARA, Czech Republic) and a transmission electron microscope (TEM, JEM-F200, JEOL, Japan) equipped with an energy-dispersive X-ray spectroscopy (EDS) detector. X-ray diffraction (XRD, X'Pert PRO, PANalytical B.V., Netherlands) patterns were recorded on a diffractometer using Cu Kα radiation. Hydrodynamic sizes and Zeta potentials were measured via dynamic light scattering (DLS) on a Zetasizer Nano ZS90 (Malvern Instruments, UK). Fourier transform infrared (FTIR) was collected using a Thermo Scientific Nicolet iN10 spectrometer. The Raman spectroscopy was measured with XploRA PLUS (HORIBA). Thermogravimetric analysis (TGA) was performed under an air atmosphere at a heating rate of 30-1000 °C/min using a TGA2 analyzer (Mettler-Toledo, Switzerland).

### 4.4 Bioinformatic and APA landscape analysis

Total RNA was extracted from RAW264.7 cells treated with either *P. gingivalis* or calf thymus DNA (500 ng/mL, 6 h). RNA-seq libraries were prepared and sequenced by BGI (Shenzhen, China) using the DNBSEQ™ platform with paired-end 150-bp (PE150) sequencing. Raw sequencing reads were subjected to quality assessment and adapter/low-quality base trimming. The resulting clean reads were aligned to the *Mus musculus* reference genome GRCm38.p6 (NCBI RefSeq assembly GCF_000001635.26) using HISAT2 (v 1.5.6). Differentially expressed genes (DEGs) were identified using Bowtie2, v 2.3.4.3. Gene Ontology (GO) enrichment and Gene Set Enrichment Analysis (GSEA) were subsequently performed. The sequencing generated approximately 6.62 Gb of clean data per sample, with 3 biological replicates per group. Bioinformatic analyses and visualization were performed using the Dr. Tom platform (BGI, https://biosys.bgi.com).

To profile the alternative polyadenylation (APA) landscape, the raw RNA-seq reads were aligned to the murine reference genome, and the DaPars2 algorithm was employed to identify dynamic APA events. A change in the Percentage of Distal poly(A) site Usage Index (|ΔPDUI| ≥ 0.2 and *p* < 0.05 were considered statistically significant.

### 4.5 Cell cultures

RAW264.7 cells was obtained from the China Center for Type Culture Collection (CCTCC, Wuhan, China; Cat. No. GDC0143). Cells were cultured in DMEM medium supplemented with 10% fetal bovine serum (FBS), 100 U/ml penicillin, and 100 μg/ml streptomycin. The cells were maintained at 37 °C in a humidified incubator with 5% CO_2_ and routinely tested to ensure they were free of mycoplasma contamination.

### 4.6 Culture of *P. gingivalis* and cell infection

*P. gingivalis* (ATCC 33277) was cultured anaerobically at 37 °C in brain heart infusion (BHI) broth supplemented with vitamin K1 (0.5 mg/l), hemin (5 mg/l), and gentamicin (50 μg/ml). Frozen bacterial stocks were revived in fresh medium and expanded under anaerobic conditions. Bacteria at the logarithmic growth phase were collected by centrifugation (8000 rpm, 5 min), washed twice with sterile PBS, and resuspended in PBS. The bacterial concentration was estimated by measuring OD600, where an OD600 of 1.0 corresponded to approximately 1 × 10^9^ CFU/ml. For infection experiments, RAW264.7 cells were incubated with *P. gingivalis* at a multiplicity of infection (MOI) of 100.

### 4.7 Cellular uptake and endosomal escape

To track the intracellular trafficking of cfDNA, calf thymus DNA (Solabio, Cat. D8020) was pre-labeled with a red fluorescent dye (Cy5.5). RAW264.7 cells were incubated with free cfDNA (Solarbio, 1 mg/ml, Cat. D8020), HMCeO_2_-PEI/cfDNA, or DA-APAT/cfDNA complexes for 2 h. Subsequently, lysosomes were stained with LysoTracker Green (Yeasen, 1:2000, Cat. 40738ES50), and nuclei were counterstained with DAPI (Yeasen, 1:1000, Cat. 708939ES08). The intracellular colocalization and endosomal escape were visualized using a confocal laser scanning microscope (Zeiss LSM 910).

### 4.8 Animals and experimental periodontitis model

Female C57BL/6 mice (8 weeks old) were purchased from WeiTong LiHua (Beijing, China) and maintained under SPF conditions. All animal procedures were approved by the Animal Care and Use Committee of the Medical Research Institute, Wuhan University (Approval No. MRI2025-LAC024).

Mice were anesthetized, and a 5-0 silk ligature was placed around the maxillary second molar to promote plaque accumulation and periodontal inflammation. Control mice received vehicle treatment alone. The ligatures were maintained throughout the experimental period. PBS, HMCeO_2_, DNase I@ HMCeO_2_ and DA-APAT solutions 10 mg/kg,10 μl per site were injected locally into the periodontal area of mice on day 1, 3 and 5 after modeling, respectively.

### 4.9 Salivary cfDNA extraction and quantification

Saliva samples were collected from female C57BL/6 mice on day 7 after experimental periodontitis induction. Following anesthesia, saliva was collected from the oral cavity, centrifuged (3000 rpm, 10 min, 4 °C) to remove debris. cfDNA was isolated from saliva samples using a commercial cfDNA extraction kit according to the manufacturer's instructions (Beyotime, D0085M). Briefly, 50 μl of saliva supernatant was processed, and cfDNA was eluted in 50 μl of elution buffer. The concentration of cfDNA was determined using the PicoGreen dsDNA assay. Purified cfDNA was incubated with PicoGreen working solution, and fluorescence was measured using a microplate reader (Ex = 480 nm, Em = 520 nm). cfDNA concentrations were calculated from a standard curve generated using calf thymus DNA standards.

### 4.10 Micro-CT analysis

Mice were euthanized on day 7 after ligature-induced periodontitis, and the maxillae were harvested and fixed in 4% paraformaldehyde for 24 h. After removal of the surrounding soft tissues, samples were scanned using a micro-computed tomography system (Bruker, Belgium) at a resolution of 10 μm (50 kV, 200 μA). Three-dimensional reconstructions were generated using NRecon software. Quantitative analyses of bone volume fraction (BV/TV), trabecular thickness (Tb.Th), and trabecular number (Tb.N) were performed within the region of interest surrounding the maxillary second molar using CTAn software. The distance from the cementoenamel junction (CEJ) to the alveolar bone crest (ABC) was measured to evaluate alveolar bone loss.

### 4.11 Statistical analysis

All quantitative data are presented as mean ± standard deviation (SD) from at least three independent experiments. Statistical significance was determined using unpaired *t*-test (for two groups) or one-way ANOVA followed by Tukey's post hoc test (for multiple groups) using GraphPad Prism (Version: 10.0). A *P*-value < 0.05 was considered statistically significant.

## CRediT authorship contribution statement

**Bin Gu:** Writing – original draft, Visualization, Methodology. **Shuyuan Qu:** Visualization, Methodology. **Yutong Wei:** Methodology. **Yilong Zhao:** Methodology. **Shuchang Hu:** Methodology. **Xiaohan Zeng:** Methodology. **Yaoting Ji:** Writing – review & editing, Project administration, Investigation, Conceptualization. **Qin Zhao:** Writing – review & editing, Supervision, Investigation, Funding acquisition, Conceptualization.

## Funding sources

This work is supported by the 10.13039/501100001809National Natural Science Foundation of China (82571092, 82372463); 10.13039/501100002858China Postdoctoral Science Foundation (2025M772606); Research Project of School and Hospital of Stomatology 10.13039/501100007046Wuhan University (ZW202502); 10.13039/501100003819Natural Science Foundation of Hubei Province (2025AFA082).

## Declaration of competing interest

The authors declare that they have no known competing financial interests or personal relationships that could have appeared to influence the work reported in this paper.

## Data Availability

Data will be made available on request.

## Abbreviations

cfDNA: Cell-free DNA
APA: Alternative polyadenylation
*P. g*,: *Porphyromonas gingivalis*
3′UTR: 3′-untranslated region
NETs: Neutrophil extracellular traps
DAMPs: Damage-associated molecular patterns
PEI: Polyethylenimine;
ROS: Reactive oxygen species
DEGs: Differentially expressed genes
GO: Gene Ontology
KEGG: Kyoto Encyclopedia of Genes and Genomes
GSEA: Gene Set Enrichment Analysis
AS: Alternative splicing
PDUI: Percentage of distal poly(A) site usage index
IGV: Integrative Genomics Viewer
XRD: X-ray diffraction
SEM: Scanning electron microscopy
TEM: Transmission electron microscopy
EDS: energy-dispersive X-ray spectroscopy
DLS: Dynamic light scattering
FTIR: Fourier-transform infrared
TGA: Thermogravimetric analysis
PD: Periodontitis
TRAP: Tartrate-resistant acid phosphatase
H&E: Hematoxylin and eosin
IF: Immunofluorescence
MFI: Mean fluorescence intensity
IHC: immunohistochemical
