# Supplementary material for: Dual-action nanotheranostics targeting cell-free DNA rescues aberrant alternative polyadenylation to ameliorate periodontitis
Source: Mater Today Bio. 2026 Oct 31;41:103776. doi: 10.1016/j.mtbio.2026.103776 (PMC13745764; doi:10.1016/j.mtbio.2026.103776)
Supplement: Supplementary file 1 [file mmc1.docx]

Supporting information

**Dual-Action Nanotheranostics Targeting Cell-Free DNA Rescues Aberrant Alternative Polyadenylation to Ameliorate Periodontitis**

Bin Gu ^a,1^, Shuyuan Qu ^a,1^, Yutong Wei ^a^, Yilong Zhao ^a^, Shuchang Hu ^a^, Xiaohan Zeng ^a^, Yaoting Ji ^a,*^, Qin Zhao ^a,*^

^a^State Key Laboratory of Oral & Maxillofacial Reconstruction and Regeneration, Key Laboratory of Oral Biomedicine Ministry of Education, Hubei Key Laboratory of Stomatology, School & Hospital of Stomatology, Wuhan University, Wuhan 430079, China.

^1^These authors contributed equally to this work

^*^Qin Zhao, Email: zhaoqin@whu.edu.cn; Yaoting Ji, Email: yaotingji@whu.edu.cn

**Figure S1.** (A) Quantitative statistics of DEGs post-cfDNA infection. (B) Volcano plot of DEGs induced by cfDNA stimulation. Log2 > 1.5, q < 0.05.

**Figure S2.** (A) Venn diagram of up-regulated genes and (B) down-regulated genes after *P. g* versus cfDNA stimulation. (C) Venn diagram showing *P. g*-related in cfDNA-DEGs.

**Figure S3.** (A) Size distribution and (B) Zeta potential measured after 1 week at 4 °C.

**Figure S4.** **Encapsulation efficiency and in vitro release kinetics of DNase I.** (A) Standard calibration curve generated for protein quantification (used for encapsulation efficiency calculation). (B) The encapsulation efficiency of DNase I within the fully assembled DA-APAT nanoplatform. (C) Standard calibration curve generated for the long-term protein release assay. (D) In vitro cumulative release profiles of DNase I from DNase I@HMCeO_2_ and DA-APAT nanoplatforms at 4 °C on days 1, 7, and 14. Data are presented as mean ± SD (n = 3).

**Figure S5.** Thermogravimetric analysis (TGA) curves of HMCeO_2_, DNase I@HMCeO_2_, and DA-APAT.

**Figure S6.** The concentration of outer/inner cfDNA.

**Figure S7.** Representative confocal fluorescence images. Nuclei were stained with DAPI (blue), cfDNA (red), lysosome (green).

**Figure S8.** Standard curve for salivary cfDNA quantification. Linear calibration curve generated using the PicoGreen dsDNA Assay with predefined DNA standards (R² = 0.999), utilized for the subsequent quantitative diagnostic readout of cfDNA in murine saliva samples.

**Figure S9.** The relative mRNA expression levels of *Il1* and *Il4*.

**Figure S10.** Representative images of immunofluorescence for HMCeO_2_-PEI and DA-APAT after *P. g* stimulation.

**Figure S11.** Relative mRNA expressions of M1/M2-relative marker. Data are presented as mean ± SD from at least three independent biological experiments unless otherwise indicated. Statistical significance was determined by *t* student test.

**Figure S12.** Relative mRNA expressions of M1/M2-relative marker after cfDNA stimulated. Data are presented as mean ± SD from at least three independent biological experiments unless otherwise indicated. Statistical significance was determined by one-way ANOVA followed by Tukey’s multiple-comparisons test.

**Figure S13.** Expression of CD86, iNOS, STING and p-STING after cfDNA stimulated.

**Figure S14.** Quantitative analysis of CEJ-ABC.

**Figure S15.** Representative H&E stained histological sections of major vital organs (heart, liver, spleen, lungs, and kidneys) collected from mice following the full DA-APAT therapeutic regimen.

**Figure S16.** Flow cytometry gating strategy for macrophage subpopulations.

**Oligo**

*Nfkbid*, proximal-sense: 5'- CGTGGGACAGTCAGCGTATG -3'; proximal-antisense: 5'- CAGAAACAATCAAGTGGGCTCG -3'; distal-sense: 5'- TGTCGTACCCTAAATCCCCA -3'; distal-antisense: 5'- ACCAAGGGGAACAATCACGG -3'.

*Sort1*, proximal-sense: 5'-AAGAAAGCAGGATGGGGGTG -3'; proximal-antisense: 5'-AGAGGGAACACACAAGAGCG -3'; distal-sense: 5'-CCCCTCTAGTCCTTTGTTCCC -3';distal-antisense: 5'-TATGGGGTCTGCCTTTGCTG -3'.

*Ctsk*, proximal-sense: 5'- GCCAGCCCATCTCTTCTCAG -3'; proximal-antisense: 5'- AATGCCCTGTTGTGTCCACA -3'; distal-sense: 5'- GTTGTGGACTCTGTGCTGGT -3'; distal-antisense: 5'- ACGCAGTGTTCTTTATTAAATTGT -3'.

*Rps6ka5*, proximal-sense: 5'- CTTGCACCTCAGCTCCCTC -3'; proximal-antisense: 5'- GCTGGCATCATGTTAGTCTGC -3'; distal-sense: 5'- ATCCCATGCGTCTTCCTGTG -3'; distal-antisense: 5'- TCCCCTCTCATATCCTTGCCT -3'.

*Eef2k*, proximal-sense: 5'- CAAGGAGAAAGGCTGGGGAA -3'; proximal-antisense: 5'- TGGAGGACGGGAAAGAGACT -3'; distal-sense: 5'- TGTGGCTGCAATTGTGTGTG -3'; distal-antisense: 5'- GGCGATGGAGCTCATGTTGA -3'.

*Ocstamp*, proximal-sense: 5'- GCACATCTCGGAGCTTCAGT -3'; proximal-antisense: 5'- CCACTTGCAGGGTCTCCATT -3'; distal-sense: 5'- ATTCACCTCGGCACTCCTCCA -3'; distal-antisense: 5'- GGCATCTTGTATTTTTATTCA -3'.

*Tgfb*, sense: 5'- CGGGAAGCAGTGCCCGAACC -3'; antisense: 5'-GGGACTCTGGATTGGACTCA -3'.

*Tnfa*, sense: 5'- CTTCTGTCTACTGAACTTCGGG -3'; antisense: 5'-CACTTGGTGGTTTGCTACGAC -3'.

*Il1b*, sense: 5'- GCAACTGTTCCTGAACTCAACT -3'; antisense: 5'- ATCTTTTGGGGTCCGTCAACT -3'.

*Il4,* sense: 5'- TGTACCAGGAGCCATATCCA -3'; antisense: 5'- CTGTGGTGTTCTTCGTTGCT -3'.

*Gapdh,* sense: 5'- AGGTTGTCTCCTGCGACTTCA -3'; antisense: 5'- CCAGGAAATGAGCTTGACAAA -3'.

**IGV visualization of APA Events**

To visually validate the dynamic (APA events at single-gene resolution, the aligned RNA-seq read files (BAM format) were first processed for depth normalization. Specifically, to ensure accurate and quantitative cross-sample comparisons, the BAM files were normalized to equivalent and converted into continuous BigWig track files using the bamCoverage tool from the deepTools suite. The normalized BigWig files were subsequently imported into the Integrative Genomics Viewer (IGV, Broad Institute, version: 2.8.9). The specific genomic coordinates corresponding to the 3UTR of the targeted macrophage polarization driver genes were precisely mapped utilizing the murine reference genome (mm10) and RefSeq gene annotations. The read coverage tracks were then dynamically scaled and visualized to explicitly delineate the differential cleavage preference at proximal versus distal PAS between the control and cfDNA-treated groups, thereby visually corroborating the quantitative PDUI shifts identified by the DaPars2 algorithm.

**In Vitro Cytotoxicity Assessment.**

The cytotoxicity of HMCeO_2_ and DA-APAT was evaluated using a Cell Counting Kit-8 (CCK-8, Solarbio, Cat. CA1210) assay. RAW264.7 cells were seeded in 96-well plates at a density of 5 × 10^3^ cells per well and incubated with different concentrations of HMCeO_2_ or DA-APAT for 24 h. Subsequently, CCK-8 working solution was added, and the cells were incubated for an additional 2 h at 37 °C. The absorbance at 450 nm was measured using a microplate reader (BioTek Synergy HTX, USA), and cell viability was expressed relative to the untreated control group.

**Evaluation of DNase I Encapsulation Efficiency and Release Kinetics**

To determine the encapsulation efficiency (EE) of the DNase I, 100 μl of DNase I stock solution was mixed with 100 μl of the DA-APAT nanoparticle suspension (1 mg/ml). The mixture was incubated overnight at 4 °C to allow for adequate loading. Subsequently, the suspension was centrifuged, and the concentration of unencapsulated DNase I in the supernatant was quantified using a standard protein assay kit (BCA Protein Assay Kit, Beyotime, Cat. P0009). The EE was calculated as the percentage of loaded protein relative to the total initial protein input.

$$EE\left（ \% \right）=\frac{C_{total}-C_{free}}{C_{total}}\times100\%$$

Furthermore, the in vitro release kinetics of DNase I were evaluated. Briefly, 20 μg of the loaded nanoparticles (DNase I@HMCeO_2_ or DA-APAT) were uniformly dispersed in 200 μl of PBS and maintained at 4 °C. At predetermined time points (days 1, 7, and 14), the samples were centrifuged, and the supernatants were collected to measure the released protein concentration. Following each collection, the nanoparticles were gently resuspended to continue the release assay.

**Quantitative Assay of cfDNA Capture and Degradation**

To quantitatively evaluate the synergistic "capture-and-degrade" capability of the nanoplatforms, three distinct material groups (bare HMCeO_2_, DNase I@HMCeO_2_, and DA-APAT) were prepared at a concentration of 1000 μg/ml. Each group was co-incubated with calf thymus cfDNA at a final concentration of 100 μg/ml in PBS at 37 °C for 2 h. Following incubation, the mixtures were centrifuged at 10,000 rpm for 10 min to separate the nanoparticle pellets from the aqueous phase.

To evaluate the capturing efficiency, the unadsorbed cfDNA remaining in the supernatant was quantified using a fluorometric dsDNA assay kit. Subsequently, to determine the enzymatic degradation efficiency, the nanoparticle pellets were collected and the residual intact cfDNA retained inside/on the materials was extracted and strictly quantified. This dual-directional quantification elucidates the dynamic clearance process of cfDNA by the nanoplatforms.

**Western Blot Analysis**

Total protein was extracted from cells using RIPA lysis buffer containing protease and phosphatase inhibitors. Protein concentrations were determined using a BCA protein assay kit (Thermo Fisher Scientific). Equal amounts of protein were separated by SDS–PAGE and transferred onto PVDF membranes. After blocking with 5% nonfat milk, the membranes were incubated with the indicated primary antibodies, P-TBK1 (Thermo Fisher Scientific, MA570165, 1:2000), TBK-1(Proteintech, 28397-1-AP, 1:2000), P-STING (Abmart, TC68060S, 1:2000), STING (Proteintech, 19851-1-AP, 1:2000), CD206 (Proteintech, 18704-1-AP, 1:2000), CD86 (ABclonal, A21198, 1:10000), iNOS (ABclonal, A3774, 1:1000), GAPDH (HUABIO, 1:6000), β-actin (ABclonal, AC026, 1:5000) overnight at 4 °C, followed by incubation with HRP-conjugated secondary antibodies (Proteintech, 1:2000, Cat. SA00001-2). Protein bands were visualized using an enhanced chemiluminescence (ECL) detection system.

**RT-qPCR Analysis**

Total RNA was extracted using TRIzol reagent (Thermo Fisher Scientific) according to the manufacturer's instructions. Complementary DNA (cDNA) was synthesized using a reverse transcription kit. Quantitative PCR was performed using SYBR Green Master Mix (Thermo Fisher Scientific, Cat. A46109) on a real-time PCR system (QuantStudio™ 1). Gene expression levels were normalized to GAPDH and calculated using the 2^-ΔΔCt method.

**Intracellular ROS Scavenging Assay**

RAW264.7 cells were seeded in 6-well plates at a density of 1 × 10^5^ cells per well and incubated overnight. Cells were treated with HMCeO_2_, DNase I@HMCeO_2_, or DA-APAT (1 μg/ml) overnight, followed by stimulation with P. gingivalis for 2 h. Intracellular ROS levels were detected using DCFH-DA fluorescent probe according to the manufacturer's instructions (Beyotime, Cat. S0034M). The fluorescence intensity was quantified by flow cytometry (Ex = 488 nm, Em = 530 nm). For fluorescence imaging, cells were treated as described above and stained with DCFH-DA, followed by nuclear counterstaining with Hoechst 33342. Fluorescence images were acquired using a fluorescence microscope.

**Flow Cytometric Analysis of Gingival Tissues**

Gingival tissues were processed into single-cell suspensions. To prevent non-specific binding, cells were first incubated with an Fc receptor blocking antibody (anti-CD16/32, Cat. ) for 15 min at 4 °C. Dead cells were excluded using Zombie Aqua viability dye (BioLegend, amcyan, 1:1000, Cat. 423101). Cells were then surface-stained with fluorochrome-conjugated antibodies against CD45 (BioLegend, APC-cy7, 1:100, Cat. 103116), CD11b (BioLegend, pBlue, 1:100, Cat. 101224), and F4/80 (BioLegend, PE-cy7, 1:100, Cat. 123113). For intracellular staining of CD86 (BioLegend, FITC, 1:50, Cat. 105006) and CD163 (BioLegend, PE, 1:100, Cat. 155308), cells were fixed and permeabilized. Compensation was performed using appropriate single-color compensation beads, and Fluorescence Minus One (FMO) controls were utilized to accurately establish gating boundaries. After exclusion of debris and, where applicable, doublets and nonviable cells, leukocytes were identified as CD45⁺ cells, and macrophages were further defined as CD45^+^CD11b^+^F4/80^+^ cells. Within the macrophage population, CD86⁺CD163⁻ cells were defined as M1-like macrophages, whereas CD86^-^CD163^+^ cells were defined as M2-like macrophages. CD86^+^CD163^+^ cells were considered a separate double-positive population and were not included in either the M1-like or M2-like populations. Flow cytometry data were acquired on a BD LSRFortessa X-20 and analyzed using FlowJo software. During analysis, debris and doublets were excluded, and viable single cells were gated for downstream macrophage subset identification.

**Histological Processing of Periodontal Tissues**

Following fixation in 4% paraformaldehyde, maxillary specimens were decalcified in 10% EDTA (pH 7.4) for 4 weeks, dehydrated, embedded in paraffin, and sectioned at a thickness of 5 μm for subsequent histological analyses.

**TRAP Staining**

Paraffin sections were stained using a tartrate-resistant acid phosphatase (TRAP) staining kit (Solarbio, Cat. G1492) according to the manufacturer's instructions. Multinucleated TRAP-positive osteoclasts were visualized by bright-field microscopy and quantified in the periodontal region.

**Hematoxylin and Eosin (H&E) and Masson's Trichrome Staining**

Paraffin sections were subjected to H&E staining to evaluate periodontal tissue morphology and inflammatory infiltration. Masson's trichrome staining was performed to assess collagen deposition and tissue remodeling. Representative images were acquired using a light microscope.

**Multiplex Immunofluorescence Staining**

Paraffin sections were deparaffinized, rehydrated, and subjected to antigen retrieval in sodium citrate buffer. After blocking with goat serum, the sections were incubated overnight at 4 °C with primary antibodies: TLR9 (Abmart, TD2970S, 1:200), CD86 (Abmart, MA8559, 1:200), and ARG1 (ABclonal, A1847, 1:200).

To visualize CD86 and ARG1 in distinct channels, appropriately cross-adsorbed secondary antibodies conjugated with different fluorochromes were applied. For instance, host, Goat anti-Mouse Alexa Fluor 488 (Thermo Fisher, 1:500, Cat. A-11001) and Host, Goat anti-Rabbit Alexa Fluor 594 (Thermo Fisher, 1:500, Cat. A-11012) were used. Nuclei were counterstained with DAPI, and images were captured using a Zeiss LSM 910 confocal microscope.

**Immunohistochemistry (IHC)**

Immunohistochemical staining was performed using primary antibodies against STING (Abclonal, Cat. A21051, 1:200). After incubation with HRP-conjugated secondary antibodies, signals were visualized using DAB substrate and counterstained with hematoxylin. Images were captured using a light microscope, and positive staining areas were quantified using ImageJ software.
